# Rare Homozygous Variants in *INSR* and *NFXL1* Are Associated with Severe Treatment-Resistant Psychosis

**DOI:** 10.3390/ijms26104925

**Published:** 2025-05-21

**Authors:** Ambreen Kanwal, Rimsha Zulfiqar, Husnain Arshad Cheema, Nauman Jabbar, Amina Iftikhar, Amina Iftikhar Butt, Sohail A. Sheikh, Jose V. Pardo, Sadaf Naz

**Affiliations:** 1 School of Biological Sciences, University of the Punjab, Lahore 54000, Pakistan; ambreen321kanwal@gmail.com (A.K.); rimshazulfiqarhere@gmail.com (R.Z.); aminaiftikhar.sbs@pu.edu.pk (A.I.B.); 2 Punjab Institute of Mental Health, Jail Road, Lahore 54000, Pakistan; husnainalvi9@gmail.com (H.A.C.); nauman.jabbar@gmail.com (N.J.); 3 Rainbow Obesity and Eating Disorders Centre, Shadman, Lahore 54000, Pakistan; aminaiftikhar7@gmail.com; 4 Psychiatry Department, Hawkes Bay DHB, Hastings 4156, New Zealand; sheik004@hotmail.com; 5 Department of Psychiatry, University of Minnesota, Minneapolis, MN 55455, USA; 6 Minneapolis Veterans Affairs Health Care System, Minneapolis, MN 55417, USA

**Keywords:** bipolar, consanguinity, exome, *INSR*, *NFXL1*, Pakistan, *RYR1*, schizophrenia

## Abstract

Psychosis constitutes a cardinal component of schizophrenia and affects nearly fifty percent of those with bipolar disorder. We sought to molecularly characterize psychosis segregating in consanguineous families. Participants from eight multiplex families were evaluated using standardized testing tools. DNA was subjected to exome sequencing followed by Sanger sequencing. Effects of variants were modeled using in-silico tools, while cDNA from a patient’s blood sample was analyzed to evaluate the effect of a splice-site variant. Twelve patients in six families were diagnosed with schizophrenia, whereas four patients from two families had psychotic bipolar disorder. Two homozygous rare deleterious variants in *INSR* (c.2232-7T>G) and *NFXL1* (c.1322G>A; p.Cys441Tyr) were identified, which segregated with severe treatment-resistant psychosis/schizophrenia in two families. There were none, or ambiguous findings in the other six families. The predicted deleterious missense variant affected a conserved amino acid, while the intronic variant was predicted to affect splicing. However, cDNA analysis from a patient’s blood sample did not reveal an aberrant transcript. Our results indicate that *INSR* and *NFXL1* variants may have a role in psychosis that requires to be investigated further. Lack of molecular diagnosis in some patients suggests the need for genome sequencing to pinpoint the genetic causes.

## 1. Introduction

A psychiatric disorder is a psychological condition in which emotions and behavioral patterns of an individual are disturbed. According to the Diagnostic and Statistical Manual of Mental Disorders 5 (DSM-5), there are multiple categories of psychiatric illnesses with diverse clinical symptoms. The most common psychiatric disorder with psychotic features (hallucinations and delusions) is schizophrenia, with a worldwide prevalence of 1%, whereas psychosis with or without other comorbidities affects 3% of people globally [1]. Bipolar disorder (i.e., manic depressive illness) is the second most common psychiatric illness affecting 1% of the world’s population; half experience psychosis in their lifetime [2]. According to twin, family and adoption studies, genetics plays a significant role in the etiology of schizophrenia and bipolar disorder with 80–85% heritability each for these disorders [3,4].

Previously, a number of genome-wide genetic linkage studies on multiplex families have revealed linkage regions on chromosomes 1q22, 5q33.2, 8p21-22, 11q23.3-24 and 20q12.1-11.23 associated with schizophrenia [5], as well as 4p16, 12q24, 18q22, 18p11, 21q21 and 22q11 with bipolar disorder [6], but these did not pinpoint the causative gene variants. Recently, the Psychiatric Genomics Consortium conducted large-scale genome-wide association studies (GWAS) for 76,755 schizophrenia cases and 243,649 controls which revealed 287 distinct loci associated with schizophrenia. Functional genomic data analysis and fine-mapping implicated 120 genes at these loci to be significantly associated with schizophrenia [7]. Another study identified rare loss of function variants in *GPR17* highly associated with schizophrenia, with high expression in the cerebral cortex [8]. Large-scale GWAS involving 41,917 affected individuals and 371,549 controls have similarly identified 64 distinct loci and 15 genes at these loci associated with bipolar disorder [9]. Exploration of rare copy-number variants in 1839 patients suffering from bipolar disorder compared to 2760 controls revealed *RNF216* significantly associated with bipolar disorder. Thus, these studies suggest that multiple genes are involved in causing bipolar disorder as well as schizophrenia, consistent with a polygenic disease model, along with some rare variant associations with these devastating illnesses [10].

Although schizophrenia and bipolar disorder are highly polygenic complex disorders, there are many examples of single rare variants associated with these illnesses in individual multiplex families, even approaching a Mendelian model. Rare missense heterozygous protein-altering variants in *LRP1B*, *PPEF2*, *GRM5* [11], *RELN* [12], *GRIN3B* [13], *TENM4* [14], *SMARCA1*, *SHANK2* [15], as well as in *ANKK1* and *ANK3* [16], have been identified as segregating with the phenotype of schizophrenia in individual multiplex families. More recently, we implicated three rare homozygous or hemizygous missense variants in *RGS3*, *IL1RAPL1* [17] and *USP53* [18] as potential causes of inherited psychoses in multiple patients in three different families. Similarly, there are also instances where predicted damaging single candidate gene variants were found segregating with the symptoms of bipolar disorder in a dominant Mendelian model, such as those affecting *KCNH7* [19], *GRID1* [20], *KANK4* [21] as well as *NRBF2*, *ANK3* and *PCDH15* [22].

The above studies indicate a continued need to search for monogenic causative genetic variants for schizophrenia and bipolar disorder. The chances of finding rare, potentially damaging genetic variants which segregate in Mendelian recessive mode of inheritance are enhanced in consanguineous nuclear families with multiple affected individuals [23]. Therefore, we followed up our previous study [24] in which we reported recruitment of families with multiple patients suffering from psychosis and included members from two additional families. Molecular genetic analyses suggested potential candidate variants for two families, and either none or ambiguous findings for the remaining participants.

## 2. Results

### 2.1. Clinical Features of the Patients

Detailed accounts of the medical histories, manifestations of the symptoms and medications for patients from families PSYAK1, PSYAK4, PSYAK5, PSYAK6, PSYAK7 and PSYAK8 have been described previously [24]. In brief, patients from these consanguineous families were suffering from psychosis (hallucinations and delusion) either as a sole symptom or in the context of schizophrenia or bipolar disorder. In addition to psychosis, 75% of the patients displayed aggressive behaviors at various times. There were two affected participants in each family. The parents and the other siblings were assessed to be unaffected.

**Figure 1:**
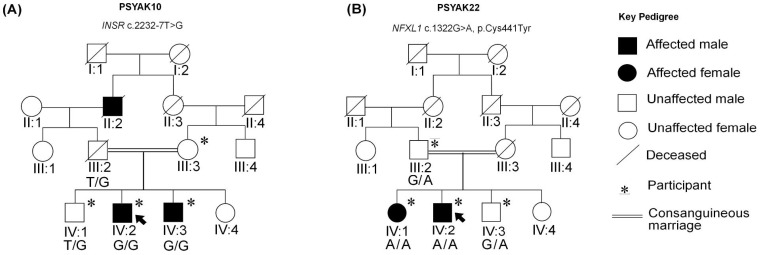
Pedigrees for families PSYAK10 and PSYAK22. (**A**) Family PSYAK10. Genotypes for *INSR* variant c.2232-7T>G are indicated below the symbols of the participants. (**B**) Family PSYAK22. Genotypes for the *NFXL1* c.1322G>A; p.Cys441Tyr variant are shown below each symbol. Arrow indicates proband.

Two male siblings and their deceased grandfather (IV:2, IV:3 and II:2) in family PSYAK10 (Figure 1A) and female and male siblings (IV:1 and IV:2) in family PSYAK22 (Figure 1B) were diagnosed with schizophrenia when they were 19, 29, 18, 20 and 23 years old, respectively. The three patients in family PSYAK10 had symptoms of severe treatment-resistant psychosis. The patients IV:2 and IV:3 were hospitalized for the last 21 and 32 years, respectively. The affected individuals were able to attend school with good grades in the initial stages of their life. Near puberty, they exhibited cognitive and behavioral deficits with intermittent angry outbursts, sadness and irritability. Symptoms common to the affected individuals included auditory and visual hallucinations (talking to imaginary objects and hearing voices of deceased people), abusive language, catatonia, paranoia, self-talking and self-smiling. They were frequently unaware of their environment. At the time of the clinical assessments for this study, the two patients were in a gross psychotic state and were unable to answer questions. Clinical histories were provided by the doctors at the hospital. These patients were unable to take care of themselves, had poor personal hygiene, and frequently spat on the floor. They could be disoriented and physically violent (Table 1). Their symptoms did not respond to the medications. Their grandfather, patient II:2, was hospitalized for 40 years before his death and was reported to have had the same symptoms.

In family PSYAK22 (Figure 1B), affected individuals IV:1 and IV:2 attended school until the ages of 12 and 13 years, respectively. Patient IV:1 developed some degree of mutism at the age of 12 years when her mother died and did not want to socialize. Her guardian reported that at the age of 18 years she displayed severe symptoms of psychosis (withdrew socially, did not engage in conversations, had false beliefs). After two years of this, she was subsequently diagnosed with negative symptoms of schizophrenia (catatonia and social withdrawal). She was unable to answer questions during the clinical assessment. Her father stated that she could perform elementary domestic chores and contribute to basic household activities. At the time of the study, she had been suffering from schizophrenia for 15 years with long intermittent periods of hospital admissions (Table 1). For patient IV:2, his father reported that the child at the age of around 5 years had severe insomnia, was physically violent, and used abusive language. He was not able to complete his education because of angry outbursts and behavioral problems. Starting at the age of 18 years, the patient would often hallucinate seeing his deceased mother. He was diagnosed with positive symptoms of schizophrenia at 23 years of age. He has been admitted continuously to the hospital for the last 10 years. At the time of the clinical assessment at the age of 34 years, he exhibited positive symptoms of schizophrenia including delusions, visual and auditory hallucinations, paranoia, self-smiling and self-talking. He was unable to provide history because of his disruptive behavior, poor insight, and gross psychotic features (low mMMSE).

### 2.2. Exome Analyses Identified Predicted Deleterious Variants in Different Genes

In family PSYAK10, a total of 22 homozygous, exonic or splice-site variants were identified with gnomAD allele frequencies less than 0.01, segregating with the phenotype (Table 2). Only a splice-site variant in *INSR* (NM_000208.4; c.2232-7T>G) was found deleterious by multiple prediction tools (Table 2). Data filtration also revealed four compound heterozygous variants in two genes segregating with the disorder (Table S2), but none of these were predicted to be deleterious.

In family PSYAK22, 12 homozygous variants (Table 2) and 14 compound heterozygous variants (Table S2) were found after filtering, which segregated with the phenotype (Table 2). Most variants either did not have deleterious supporting scores or lacked evolutionary conservation of the affected amino acids (Table 2). Only a missense variant of *NFXL1* (NM_001278624.2; c.1322G>A; p.Cys441Tyr) had high deleterious scores according to multiple prediction tools, affected an evolutionary conserved amino acid, and also implicated a gene which had high expression in the brain. None of the identified shared compound heterozygous variants were predicted to be deleterious (Table S2).

Filtration of exome data for families PSYAK1, PSYAK4, PSYAK5, PSYAK6, PSYAK7 and PSYAK8 revealed various homozygous/hemizygous variants (Table S3). Multiple compound heterozygous variants were also detected for families PSYAK1, PSYAK4, PSYAK6 and PSYAK8 (Table S4), but none for families PSYAK5 and PSYAK7. Most of the variants detected in the different families were predicted to be non-detrimental to splicing, were also homozygous/hemizygous in numerous controls, and/or did not affect amino acids conserved in different orthologues. A few exceptions were observed for two families as detailed below. In family PSYAK5, a homozygous variant of *CRYBB3* c.470C>T, p.(Thr157Met) was predicted deleterious by multiple software (Table S3), but the affected amino acid was not conserved among different orthologues (UCSC genome browser). The patients also had a homozygous variant of *DOCK1* c.814A>C, p.(Ile272Leu) which affected a conserved amino acid (https://genome.ucsc.edu/; accessed on 1 March 2024) but was predicted deleterious by only a few software programs (Table S3).

**Figure 2:**
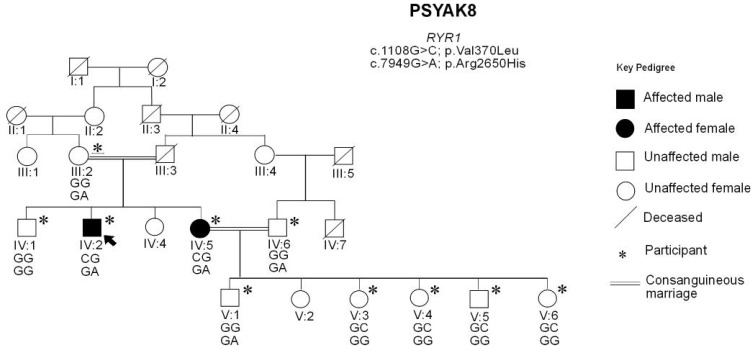
Updated pedigree of PSYAK8. The family was previously presented [24]. However, individuals IV:6, V:1 and V:3 to V:6 were newly recruited for this study. Two patients were diagnosed with psychotic bipolar disorder. Genotypes for *RYR1* c.1108G>C and c.7949G>A variants are mentioned below the symbol of each participant. The paternal haplotype is shown first in each case. Only two patients had both *RYR1* variants present together in trans.

In family PSYAK8, two pairs of compound heterozygous variants (Table S4) segregated with the phenotype. Only one of the variants, *RYR1* p.(Arg2650His) was predicted to be deleterious and had high damaging scores. The second *RYR1* p.(Val370Leu) variant was predicted to be benign. It also affected an amino acid which was not conserved in evolution. Subsequent genotype analyses for *RYR1* included five newly recruited unaffected children of patient IV:5 (Figure 2) and further supported the segregation of the variants with the phenotype. *RYR1* variants have previously been associated with bipolar disorder [25].

### 2.3. Multiple Regions of Shared Homozygosity Were Detected for All Families Except in Family PSYAK1

No region on any chromosome was identified which was homozygous only in the patients and heterozygous in the unaffected individuals for family PSYAK1 (Figure S1). Multiple regions of homozygosity shared by affected individuals were identified in families PSYAK4 PSYAK5, PSYAK6 and PSYAK7 (Figures S2–S5). Only one region of homozygosity was identified for the patients of family PSYAK8 (Figure S6). However, no predicted deleterious variants were found in these regions of homozygosity for any of the patients in these families (Table S3).

**Figure 3:**
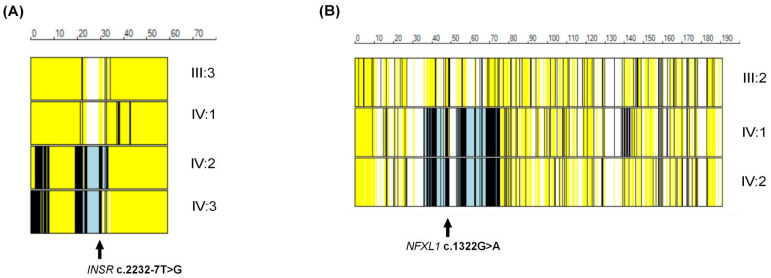
AgileVCFMapper displays for families PSYAK10 and PSYAK22. (**A**) AgileVCFMapper display for chromosome 19 of family PSYAK10. IV:2 and IV:3 are affected individuals, IV:1 is an unaffected sibling, and III:3 is the unaffected mother. Each individual chromosome is represented by a horizontal box in which yellow bars indicate heterozygous calls, black bars denote homozygous calls and long, uninterrupted black/gray segments signify regions of autozygosity or homozygosity. (**B**) AgileVCFMapper display for chromosome 4 of family PSYAK22. Individuals IV:1 and IV:2 are affected, and III:2 is the unaffected father.

Autozygosity mapping of exome data of all members of families PSYAK10 and PSYAK22 also revealed multiple regions of homozygosity (Figures S7 and S8). Variants *INSR* (c.2232-7 T>G) and *NFXL1* (c.1322G>A) were identified in the regions of homozygosity on chromosomes 19 and 4 in the respective families (Figure 3A,B).

### 2.4. The Predicted Deleterious Variants Segregated with the Phenotypes

**Figure 4:**
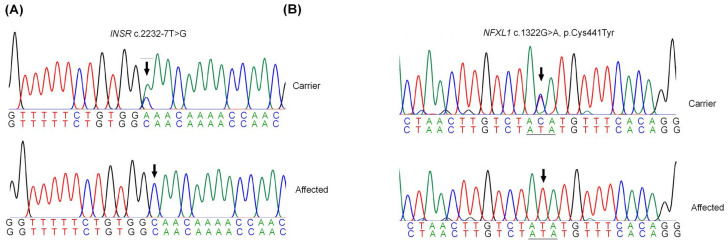
Sequence traces for families PSYAK10 and PSYAK22. (**A**) Partial electropherograms for family PSYAK10. DNA sequence traces of *INSR* c.2232T>G for the heterozygous mother and the homozygous affected individuals. Arrow indicates the position of variant. Reverse complement sequences are shown. (**B**) Partial electropherograms for *NFXL1* variant. Sequence traces of *NFXL1* c.1322G>A for the heterozygous father and the homozygous affected individuals. Reverse complement sequences are shown.

Sanger sequencing confirmed the segregation of *INSR* and *NFXL1* variants in the respective families since the obligate carriers and the unaffected siblings were heterozygous while only the patients were homozygous (Figure 4A,B). The aggregated allele frequencies in the public gnomADv4 database for *INSR rs775596300* and *NFXL1 rs748118226* variants were 0.0000717 and 0.000003985, respectively, with none homozygous (accessed on 1 March 2024).

### 2.5. The Predicted Deleterious Alleles Are Absent in the Ethnically Matched Population

We did not detect *INSR* and *NFXL1* variants in 102 and 122 unrelated ethnically matched controls, respectively, after allele-specific PCR or within the in-house exome data of 300 unrelated ethnically matched individuals, supporting the rarity of these variants in the Pakistani population. In-house exome data of 300 individuals also lacked the shortlisted compound heterozygous *RYR1* variants. However, although the public database gnomADv4 allele frequency of *RYR1* p.(Val370Leu) variant was 0.0000369 (accessed on 1 March 2024), allele-specific PCR on 122 ethnically matched controls detected seven individuals heterozygous for the *RYR1* p.(Val370Leu) variant (>0.02 allele frequency), which indicates that it is a common allele in our local population. We did not detect the *RYR1* p.(Arg2650His) variant in any of our ethnically matched controls, although it is present in the public databases with a gnomADv4 allele frequency of 0.0000143 (accessed on 1 March 2024).

### 2.6. The Candidate Genes Are Expressed in the Brain

The Human Brain Transcriptome database (https://hbatlas.org/pages/hbtd; accessed on 1 January 2025) shows high expression of *INSR* [26], *NFXL1* [27] and *RYR1* [28] in adult and developing human brains (Figures S9–S11). The Human Protein Atlas (https://www.proteinatlas.org/; accessed on 1 January 2025) revealed a high level of INSR (Figure S12), NFXL1 (Figure S13) and RYR1 (Figure S14) in the human brain, especially in the regions of white matter, the cerebellum and the hypothalamus.

### 2.7. Insulin Receptor (INSR) c.2232-7T>G Variant

**Figure 5:**
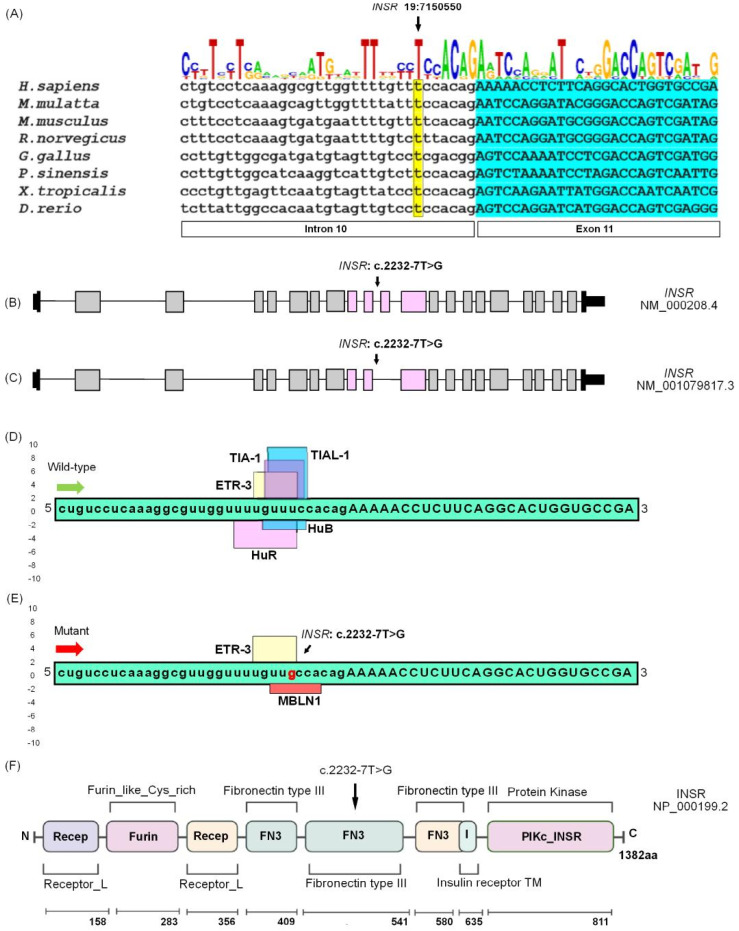
INSR conservation, isoforms, predicted splicing effects and protein domains. (**A**) Evolutionary conservation of *INSR* intronic nucleotide c.2232-7T>G. A comparison is shown for eight diverse representative orthologues from all vertebrate groups. The vertical dimension of each letter in the consensus sequence above the nucleotide sequence is proportional to the frequency of the corresponding nucleotide in the analyzed orthologues. The exonic nucleotides are denoted by the capital letters, and the intronic nucleotides are represented by the lowercase letters. The yellow-highlighted nucleotide indicates the position of the variant. Analysis indicates that the c.2232-7T position is fully conserved. (**B**) *INSR* isoform (NM_000208.4) encodes a protein with a molecular weight of 1382 amino acids and consists of 22 exons. The arrow indicates the position of the variant identified in family PSYAK10. Black boxes represent the exons that are non-coding, while empty boxes represent the coding exons. Horizontal lines are used to illustrate the introns. (**C**) *INSR* isoform (NM_001079817.3) contains only 21 exons. (**D**) SpliceAid predicted that ESEs (exonic splicing enhancers) and ISSs (intronic splicing silencers) within the wild-type pre-mRNA of *INSR* will interact with the splicing proteins ETR-3, TIA-1, TIAL-1, HuR and HuB. Green and red arrows indicate the wild-type and the mutated sequences of the pre-mRNA, respectively. (**E**) The same program predicted only ETR-3 and MBLN1 proteins interacting with the mutant pre-mRNA *INSR* due to the variant c.2232-7T>G. (**F**) Domain structure of INSR. Variant c.2232-7T>G will potentially affect the fibronectin type III (FN3) domain, which forms an immunoglobulin-like fold spanning amino acids 462 to 541.

The *INSR* variant c.2232-7T>G affects an absolutely conserved intronic site (Figure 5A). Two isoforms exist for *INSR*. The longest isoform of *INSR* (NM_000208.4) consists of 22 exons. The variant detected in family PSYAK10 is in intron 10 (Figure 5B). The other isoform (NM_001079817.3) consists of 21 exons, as it lacks translated sequences corresponding to the 11th exon of the longer isoform (Figure 5C). In silico analysis for the *INSR* isoform (NM_000208.4) shows no significant impact on splicing as a result of the c.2232-7T>G variant, whereas significant alteration of RNA splicing was predicted due to disruption of exonic splicing enhancers (ESEs) and exonic splicing silencers (ESSs) for the *INSR* isoform (NM_001079817.3). The Berkeley Drosophila Genome Project predicted the intronic variant to significantly reduce the splicing of both isoforms. The SpliceAid tool predicted that during post-transcriptional RNA processing, five proteins likely interact with the wild-type sequence, out of which three proteins act as exonic splicing enhancers (ESEs) and two as intronic splicing silencers (ISSs), whereas only two proteins interact with the mutant *INSR* sequence (Figure 5D–E). This could significantly reduce the efficiency of splicing.

The full length INSR protein (UniProt ID: P06213) possesses multiple domains. The variant detected in the affected individuals in family PSYAK10 is present in an intron preceding an exon encoding a fibronectin type III domain (Figure 5F). This domain forms a beta sandwich structure and is responsible for binding different ligands to INSR [29].

### 2.8. The INSR c.2232-7T>G Variant Did Not Affect Splicing of RNA Obtained from Blood

The sequencing of recombinant plasmids from six colonies obtained after cloning *INSR*-specific cDNA from a patient’s blood sample, did not reveal any aberrant transcripts. Any effect of the variant on splicing, specifically in the brain, or other potential ways the variant may affect the *INSR* transcripts, remains to be explored.

### 2.9. Nuclear Transcription FACTOR, X-Box Binding-like 1 (NFXL1) c.1322G>A; p.(Cys441Tyr) Variant

**Figure 6:**
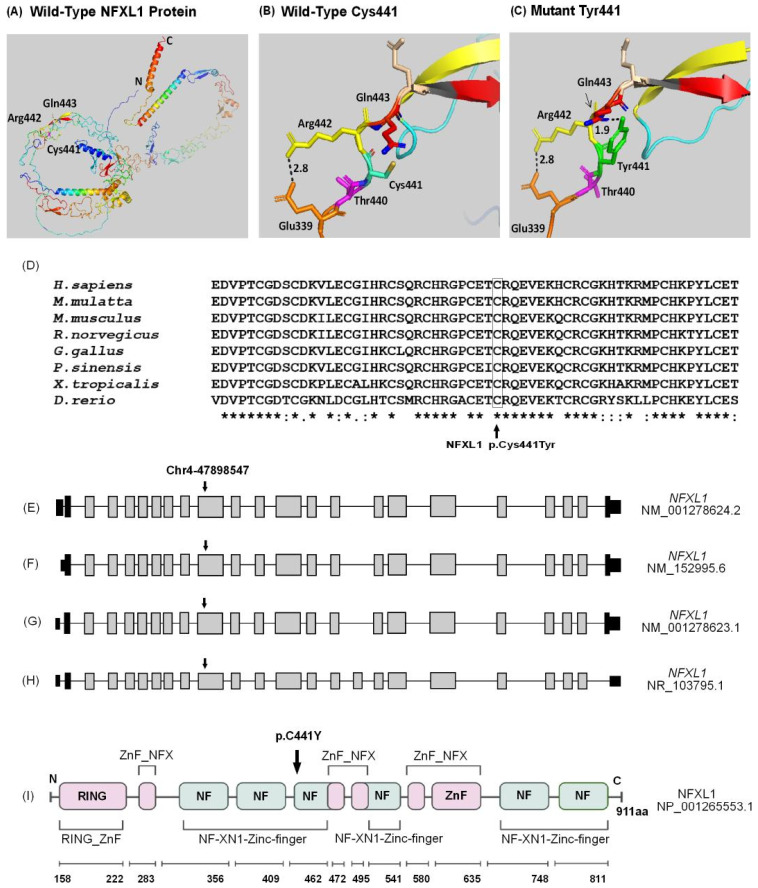
Structural modeling, evolutionary conservation, isoforms and protein domains of NFXL1. (**A**) Structural model of wild-type NFXL1 protein representing N-terminal and C-terminal, as well as the amino acids p.Cys441, p.Arg442, and p.Gln443. (**B**) Wild-type NFXL1 p.Cys441 forms covalent bonds with p.Thr440 and p.Gln443. (**C**) Mutated NFXL1 p.Tyr441 resulted in the formation of an additional interacting bond with p.Gln443, with a bond length of 1.9 A⁰ (shown by an arrow). (**D**) Multiple sequence alignment of NFXL1 displaying the conservation of cysteine 441 amino acid in human, monkey, mouse, rat, chicken, turtle, frog and fish. “*” Absolutely conserved amino acids, “:” Conservative amino acid change, “.” Amino acid conserved in some species. (**E**) *NFXL1* isoform (NM_001278624.2) encodes a protein with 911 amino acids. The arrow denotes the position of the variant detected in family PSYAK22. Black boxes indicate the non-coding exons, empty boxes represent the coding exons, and the black lines denote the introns. (**F**) *NFXL1* isoform (NM_152995.6) contains 23 exons. (**G**) *NFXL1* isoform (NM_001278623.1) with the variant c.1322G>A within exon 10. (**H**) *NFXL1* isoform (NR_103795.1) contains 24 exons. (**I**) Schematic representation of NFXL1 isoform encoded by NM_001278624.2, showing multiple domains including RING-type zinc finger, NF-XN1-type zinc finger and NF-X-type zinc finger domains. The c.1322G>A, p.(Cys441Tyr) variant is within the NF-XN1-type zinc finger domain, which is involved in DNA binding.

The three-dimensional structure of the wild-type NFXL1 protein structure from Alpha-fold (UniProt ID: Q6ZNB6) suggests that the p.Cys441 resides on an α-helix, with a confidence score of 86.2% (Figure 6A). p.Cys441 is not predicted to form ionic interactions with other neighboring amino acids, including p.Thr440, p.Arg442 and p.Gln443 (Figure 6B). Substitution of Tyr at this position p.(Cys441Tyr) results in the formation of a new hydrogen bond with p.Gln443, with a bond length of 1.9 A^0^ (Figure 6C). The variant NFXL1 p.(Cys441Tyr) affects an amino acid which is highly conserved among different orthologues (Figure 6D). *NFXL1* isoform (NM_001278624.2) consists of 23 exons, and the variant identified in the family PSYAK22 is in exon 10 (Figure 6E). There are four other isoforms as well (Figure 6F–H). The protein NFXL1 (UniProt ID: Q6ZNB6) has multiple conserved domains, with the variant affecting the NF-XN1-type zinc finger domain (Figure 6I). The NF-XN1-type zinc finger domain is a zinc binding domain which is predicted to facilitate the function of DNA-binding transcription factors, specifically those that interact with RNA polymerase II in a sequence-specific manner [30].

PredictProtein indicated that p.Cys441 is present in a highly conserved (conservation score = 7–9, high) buried region of the protein with limited solvent accessibility (B-value = 30–70). I-Mutant Suite showed a large decrease in protein stability due to the p.Cys441Tyr variant (reliability index = 1). SNAP2 suggested the p.(Cys441Tyr) variant as strongly unfavorable to the function of the protein (score = 90). CUPSAT predicted that this variant destabilizes the torsion angle and is unfavorable with a ΔΔG value of −5.37 kcal/mol. Analysis by HOPE revealed that the mutant residue is bigger, which might cause bumps in the protein and disturb hydrophobic interactions of the protein (Variant’s MetaRNN score is 0.988 = pathogenic) [31].

### 2.10. Ryanodine Receptor 1 (RYR1) p.Val370Leu and p.Arg2650His Compound Heterozygous Variants

**Figure 7:**
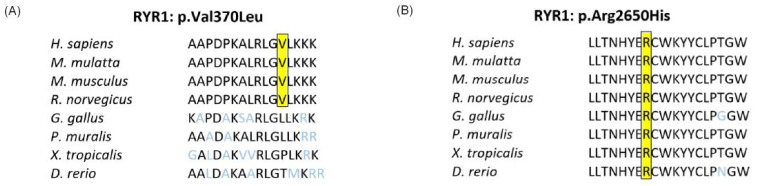
Evolutionary conservation of amino acids affected by the variants in family PSYAK8. (**A**) Multiple sequence alignment for RYR1 p.Val370 among diverse orthologues. Valine is conserved in mammals, while other species possess leucine, proline or threonine as the wild-type sequence. (**B**) Clustal Omega sequence alignment of RYR1 p.Arg2650 conservation from diverse vertebrates. Arginine is conserved in all vertebrate orthologues, with the exception of *A. spinifera* (softshell turtle), where it is substituted with glutamine (not shown in this alignment). Note that histidine as found in PSYAK8 patients in this position has different properties from glutamine. Yellow highlights indicate the amino acids of RYR1 affected by the variants in the family.

**Figure 8:**
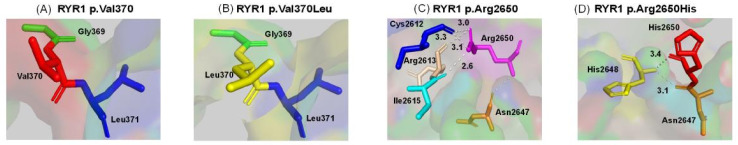
Structural modeling of RYR1 variants in family PSYAK8. (**A**) Structural model of wild-type RYR1 protein showing p. Gly369, p.Val370 and p.Leu371 amino acids in which p.Val370 did not form any polar interactions with other amino acids. (**B**) Replacement of valine by leucine in RYR1 p.(Val370Leu) also did not affect any of the polar interactions of the wild-type or mutated amino acid. (**C**) Wild-type RYR1 p.Arg2650 forms four polar interactions with p.Cys2612, p.Arg2613, p.Il12615 and p.Asn 2647. (**D**) Mutated RYR1 p.(Arg2650His) forms only two polar bonds with p.Asn 2647 and p.His2648.

The RYR1 Val370 residue was only conserved in mammals, whereas the Arg2650 residue was conserved among all the diverse orthologues (Figure 7A, B). Structural modeling of the wild-type RYR1 protein (UniProt ID: P21817) revealed that the residues p.Val370 and p.Arg2650 reside in the exposed and buried positions, respectively (Figure 8A–D). PredictProtein revealed that p.Val370 is located within a loop and p.Arg2650 is situated within an α-helix. It further predicted that the RYR1 arginine variant is in a buried region of the protein, whereas valine is in an exposed region of the protein with high accessibility to solvent molecules. I-Mutant Suite predicted that p.Val370Leu substitution causes only a slight decrease in the stability, whereas the p.Arg2650His variant greatly reduces the stability of the protein. The functional prediction provided by SNAP2 showed the p.Val370Leu variant as neutral, with a score of −94 and an accuracy of 97%, whereas the p.Arg2650His variant was highly unfavorable with a score of 43 and an accuracy of 71%. CUPSAT suggested that the torsion angles created by the p.Val370Leu and the p.Arg2650His variants are essentially unfavorable to the protein structures, with a ΔΔG value of 0.56 kcal/mol and 0.81kcal/mol, respectively.

**Figure 9:**
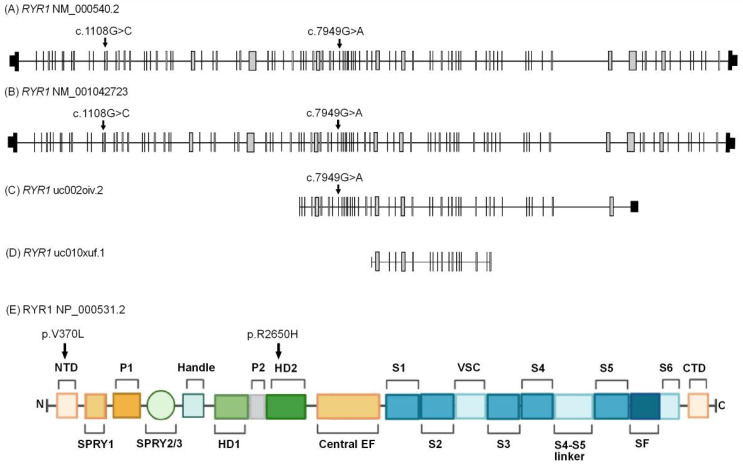
Isoforms and domains of RYR1. (**A**) *RYR1* isoform (NM_000540.2) encodes the highest molecular weight protein, consisting of 5038 amino acids. A black box indicates non-coding exons, and arrows mark the position of the compound heterozygous variants detected in family PSYAK8. (**B**) *RYR1* isoform (NM_001036188.1) contains 106 exons with the variant c.1108G>C within exon 11 and variant c.7949G>A within exon 50. (**C**) *RYR1* isoform (uc002oiv.2) encodes a truncated protein. (**D**) *RYR1* isoform (uc010xuf.1) is the shortest isoform. (**E**) Schematic representation of RYR1 isoform (NP_000531.2) showing multiple domains. The c.1108G>C, p.(Val370Leu) variant is within the N-terminal domain (NTD), and the c.7949G>A, p.(Arg2650His) variant is within helical domain 2 (HD2) of RYR1 [33].

*RYR1* isoform (NM_000540.2) comprises 106 exons. The variants c.1108G>C and c.7949G>A are located within exons 11 and 50, respectively (Figure 9A). Alternative splicing gives rise to four additional isoforms (Figure 9B–D). RYR1 possesses multiple evolutionarily conserved domains, including the helical domain which contains phosphorylation sites for protein A (Figure 9E). RYR1 serves an integral function in neuronal calcium signaling dynamics by releasing calcium from its intracellular stores [32].

## 3. Discussion

We describe molecular genetic findings from sixteen patients exhibiting severe forms of psychoses from eight different families. Biallelic variants in *INSR*, *NFXL1* and *RYR1* were identified in affected individuals of three families, while we could not pinpoint predicted deleterious variants for patients in five families. Taken together with our previous results [17,18] (Figure S15), and excluding *RYR1*, we have potential candidates for patients in five families with psychosis. This indicates that even in highly consanguineous families where multiple variants may be homozygous in offspring and seem to segregate with the disorder by chance alone, this is not actually the case. Either no homozygous deleterious variant was identified at all, or only one homozygous or hemizygous deleterious variant was correlated with the phenotype, indicating the importance of rare variants as possible causes of psychotic disorders. On the other hand, the lack of clear molecular diagnosis for six families may indicate that many cases of psychoses are polygenic or are due to variants undetected by exome sequencing.

The gene for the insulin receptor (OMIM#147670), also known as *HHF5* or *CD220*, is located on chromosome 19p13.2. It encodes a protein of 1382 amino acids which consists of multiple functional domains including fibronectin type III-1, fibronectin type III-2, fibronectin type III-3 and a protein kinase domain (Figure 5F). INSR is encoded as a single-chain polypeptide precursor which, upon post-translational modification, forms α- and β-subunits. Mature INSR is heterotetrameric, with two extracellular α-subunits and two transmembrane β-subunits which together function in ligand binding and intrinsic tyrosine kinase activity responsible for signal transduction [34].

INSR is encoded by two alternatively spliced isoforms, distinguished by the presence (Figure 5B; isoform B) or absence (Figure 5C; isoform A) of a 36-nucleotide exon 11 inserting 12 amino acids in the fibronectin domain [35]. In vitro experiments have indicated that the length of the polypyrimidine tract at the 3′ end of intron 10 (including position c.2232-7T>G) causes alternative splicing of exon 11 [36]. Additional nucleotides in exon 11 within the promoter region of *INSR* have also been found to exert an influence on alternative splicing [36]. Although predicted to affect splicing, the variant c.2232-7T>G was not seen to affect *INSR* in analysis of cDNA obtained from a patient’s blood sample. It is possible that the variant could reduce the amount of isoform-specific mRNA, without affecting splicing, as has been reported before [37]. Another possibility is that the variant may result in large-sized intronic/exonic retentions within aberrant transcripts, which would not be captured by our analysis because of the experimental design. Assays using exon-trap systems or studying splicing after engineering the variant into the genome of an appropriate cellular or organoid system may help determine if the variant effects splicing or changes the levels of different isoforms.

There is evidence supporting the role of *INSR* variants in psychiatric disorders. Sixteen variants in *INSR* have been reported to be associated with autism spectrum disorder (HGMD, accessed on 1 April 2025). *INSR* SNPs *rs2229431* and *rs12610022* were found to be significantly different in allele frequencies and genotypic distributions in schizoaffective disorder patients as compared to the controls [38]. In another study, *INSR* SNPs *rs747721248* and *rs2229431* were significantly associated with schizophrenia [39]. *INSR* expression level is also affected in other brain disorders such as in patients with Alzheimer’s disorder [40].

The role of INSR in brain health has been shown in model organisms as well. In zebrafish, *Insr* is expressed in neurons and neural stem cells where it plays a role in neurogenesis [41]. Hippocampal-specific deletion of *Insr* impairs episodic memory and spatial memory in engineered mice [42]. In another model mouse, anxiety symptoms were noted after reducing *Insr* expression in glutamatergic and GABAergic neurons [42]. In a separate study, lentivirus-mediated downregulation of hypothalamic *Insr* caused anhedonia, depressive-like and anxiety-like behaviors in rats [43].

Nuclear Transcription Factor, X-Box Binding-Like 1 *NFXL1* (OMIM#620488), also known as *HOZFP*, *URCC5*, *OZFP* or *CDZFP*, is located on chromosome 4p12 and encodes a protein of 911 amino acids. NFXL1 is a DNA-binding transcription factor. It has zinc finger domains which facilitate the binding to DNA and a RING domain which functions as an E3 ubiquitin ligase [44]. The expression level of this particular transcription factor has been demonstrated to be significantly elevated in embryonic stem cells before they differentiate into myelinated oligodendrocytes. Additionally, it exhibits an increased level of expression during the early stages of mouse embryonic development (E11.5) and in the cerebellar structures in humans. *NFXL1* has been reported to play a role in regulating the NFĸB pathway [44], whose dysregulation is already known to cause neuroinflammation in a subset of schizophrenia patients [45].

Ten heterozygous *NFXL1* missense/nonsense variants have been associated with various neurological disorders which include specific language impairment, speech delay and intellectual disability. Among these, different heterozygous nonsynonymous variants in *NFXL1* were identified to cause specific language impairment in a founder population from Robinson Crusoe Island [44]. Four missense variants p.(Cys312Arg), p.(Trp359Cys), p.(Gln583=) and p.(Arg815His) were associated with autism spectrum disorder [46] (HGMD, accessed on 1 April 2025). *NFXL1* is highly expressed in the human cerebellum, which is known to play a significant role in cognitive function and in language development [47]. *Nfxl1* knock-out mice have decreased body weights and body lengths along with skeletal abnormalities (https://www.informatics.jax.org/, accessed on 1 March 2024). However, behavioral defects remain unexplored.

Ryanodine receptor 1 *RYR1* (OMIM#180901), also known as *PPP1R137*, *RYR-1* and *RYDR*, is located on chromosome 19q13.2 and encodes a protein of 5038 amino acids. RYR1 functions by releasing calcium ions from intracellular stores in neuronal cells and is known to promote extended calcium signaling in the brain [32]. The full length protein contains multiple evolutionary conserved domains including N-terminal domain (NTD), SPRY1 domain, two helical domains, central domains, a transmembrane domain with ion pore, and a C-terminal domain [33] (Figure 9E). Multiple variants of *RYR1* cause disorders such as congenital myopathy 1B, autosomal recessive (OMIM#255320); the dominantly inherited King Denborough syndrome (OMIM#619542); as well as congenital myopathy 1A, autosomal dominant, with susceptibility to malignant hyperthermia and central core disease (OMIM#117000). Some *RYR1* de novo variants, such as p.(Arg3672His), p.(Asn4036Asp), and p.(Thr5005Ala), have been found to be associated with neurological disorders including autism spectrum disorder [46].

Many genes associated with bipolar disorder encode calcium channel subunits [48]. GWAS have also implicated calcium channel regulation as one of the major pathways involved in genetic predisposition to bipolar disorder [48]. Genes encoding calcium channels, such as *RYR1* and *CACNA1D*, have been identified as risk loci for bipolar disorder due to their roles in calcium signaling and the regulation of circadian rhythms [25]. Some studies have also associated RYR1 protein expression in regulating depression-like behaviors in mouse models [49].

In family PSYAK8, we detected a predicted deleterious variant of *RYR1*, while the second variant was not supported as deleterious, and moreover, our experiments determined it to be a polymorphism. There are several cases where otherwise non-damaging variants, including polymorphisms, can cause a disease when present in trans with a deleterious allele. For example, compound heterozygous *ABCA13* variants p.(His3609Pro) and p.(Thr4550Ala) were correlated with the phenotype in three patients suffering from bipolar disorder [50]. Both of these variants impact partially-conserved amino acids, with some mammalian or reptilian species orthologues having the Pro or Ala residues at the respective sites of the wild-type sequences [50]. A second instance is that of a heterozygous 200 kb deletion in *RBM8A* causing thrombocytopenia-absent radius-TAR syndrome (OMIM#274000) only when inherited in trans with a heterozygous c.-21G>A common polymorphism [51]. This suggests that the *RYR1* compound heterozygous variants detected in the current research require further study to establish their link to the phenotype.

Interestingly, mice with selective reduced expression of *Ryr1* in the cerebral cortex and hypothalamus display impaired behavioral capabilities [52]. Repeated administration of oligonucleotides that are antisense to the *Ryr1* sequence resulted in an antidepressant-like response in mice, evidencing the significance of RYR1 as a crucial target in the context of mood disorders [53]. *Ryr2* and *Ryr3* knockdown mice also display memory deficits [53]. *Ryr3* knockout mice have symptoms of social isolation and excessive hyperactivity [49,54].

Our research indicates a need for further evaluation of *INSR*, *NFXL1* and possibly *RYR1* in psychosis in humans. However, there are notable limitations in our studies. First, though the detected variants are potentially highly penetrant and likely pathogenic for psychosis, they cannot be definitively asserted as monogenic causative variants. This is due to the possibility of combined effects of multiple common variants (>1% allele frequency) contributing to the disease in patients in the presence of these high-risk alleles. Second, these variants could be specific to individual families and may not be replicated in future studies. Third, due to a lack of funding, the pathogenicity of the detected variants was not experimentally demonstrated, which is an important step in establishing a causal link between the genetic variant and the observed phenotype. Lastly, other types of genetic variations, such as copy number variants, repeat expansions or intragenic variants, cannot be ruled out as the underlying cause of the disorders. Therefore, we suggest leveraging emerging technologies, such as long-read whole-genome sequencing in multiplex families to delineate the underlying causes. However, notwithstanding these limitations, we believe that it is unlikely to be the case that every detected variant and finding is by chance alone. This is further strengthened by the fact that the deleterious variants were detected in genes expressed in the brain which have previous correlations with various neurological disorders, including psychiatric symptoms in some cases.

## 4. Materials and Methods

### 4.1. Ethical Approval and Recruitment

The study was completed according to the guidelines and regulations of the institutional review boards at the School of Biological Sciences, University of the Punjab, Lahore, Pakistan (IRB# 00005281, FWA 00010252) and the University of Minnesota, Minneapolis, USA (FWA00000312). Detailed information regarding recruitment procedures and the clinical tests for six families PSYAK1, PSYAK4, PSYAK5, PSYAK6, PSYAK7 and PSYAK8 has been reported previously [24], though five new unaffected individuals of family PSYAK8 were also inducted into the current study (see below). Two families, PSYAK10 and PSYAK22, were newly identified by visiting psychiatric wards of a local hospital in Lahore. Participants were recruited by written informed consents. Clinical testing was performed using Diagnostic Interview for Genetic Studies (DIGS) [55], modified MiniMental Status Examination (mMMSE) [56], Hamilton Depression and Anxiety Rating Scales (HAM-D, HAM-A) [57], Positive and Negative Syndrome Scale (PANSS) [58], and Positive and Negative Syndrome Scale (PANSS) [59] and sampling were completed (Supplementary Methods). 

### 4.2. Exome Sequencing

Exome sequencing was performed on DNA of twelve previously enrolled [24] and four new patients affected with psychosis in eight families. Samples from available unaffected siblings and unaffected parents were also included. The sequenced samples were from 34 participants: 16 patients (9 males and 7 females), with 11 samples from the unaffected parents and 7 unaffected siblings of the patients (4 males and 3 females). Exome sequencing data were generated using 150-bp paired-end read length with 100X depth and aligned to the Human GRCh37/hg19 assembly.

### 4.3. Variant Analyses of the Exome Data

We uploaded the exome variant call files (VCF) to the Franklin website (https://franklin.genoox.com/; accessed on 1 March 2024) and annotated them after applying standard filters (Supplementary Methods). Homozygous and compound heterozygous variants were selected. Priority was given to the variants with the highest deleterious scores from multiple pathogenicity prediction tools (Supplementary Methods). We also located all autosomal regions homozygous in the patients’ data due to identity by descent. For this purpose, the VCF data were separately uploaded to AgileMultideogram software (http://www.dna-leeds.co.uk/agile/AgileVCFMapper/; accessed on 1 March 2024) and AgileVCFMapper (https://dna-leeds.co.uk/agile/; accessed on 1 March 2024) [60] for analyses (Supplementary Methods).

### 4.4. Segregation Analyses and Allele Frequencies in the Local Population

The segregation of each variant was checked in all the participants from each family by performing gene-specific amplification and Sanger Sequencing. Primers were designed with Primer3 (https://bioinfo.ut.ee/primer3-0.4.0/; accessed on 1 March 2024) (Table S1). The genomic sequences of *INSR* and *NFXL1* corresponding to NM_000208.4 and NM_001278624.2, respectively, were obtained. Big Dye Terminator V3.1 kit (ABI Thermo Fisher Scientific, Waltham, MA, USA) was used for Sanger sequencing, and electrophoresis was completed on an ABI3730 sequencer. Primers (Table S1) for competitive allele-specific [61] and Tetra primer ARMS [62] PCR was designed using Primer1 (http://primer1.soton.ac.uk/primer1.html; accessed on 1 March 2024), and reactions were performed in standard conditions (Supplementary Methods). Additionally, allele frequencies of all filtered variants were also checked in the in-house exome data of 300 unrelated ethnically matched individuals.

### 4.5. Nucleotides and Amino Acids: Conservation and Modeling

The conservation of the nucleotides affected by the splice-site variants was observed by accessing genomic sequences of orthologues on the UCSC genome browser. If conservation was suspected, the genomic sequences of all the diverse orthologous genes were retrieved from the respective genome assemblies available on the UCSC genome browser. These sequences were aligned using Clustal Omega (https://www.ebi.ac.uk/Tools/msa/clustalo/; accessed on 1 March 2024) with the “DNA” option. The region with the splice variant was identified from the generated alignment by using a simple search function. The corresponding aligned sequences were uploaded at the WEBLOGO site (https://weblogo.berkeley.edu/logo.cgi; accessed on 1 March 2024) to generate a graphical representation, in which each nucleotide was assigned a specific height based on the degree of the conservation. For conservation of amino acids, protein sequences were retrieved from UniProt (https://www.uniprot.org/; accessed on 1 March 2024), and the amino acid orthologous sequences were aligned using the Clustal Omega Sequence Alignment Tool (https://www.ebi.ac.uk/Tools/msa/clustalo/; accessed on 1 March 2024).

The three-dimensional structure of the wild-type NFXL1 and RYR1 proteins were taken from the AlphaFold Protein Structure Database (https://alphafold.com/; accessed on 1 March 2024) using the UniProt IDs Q6ZNB6 and P21817, respectively. The variants NFXL1 p.(Cys441Tyr); RYR1 p.(Val370Leu); p.(Arg2650His) were introduced into the wild-type protein sequences in PyMoL using the site-directed mutagenesis program option, and non-polar contacts to any other atoms were found for the wild-type and the mutated amino acids (Supplementary Methods).

### 4.6. Isoforms and Domains Analyses for Different Genes and Encoded Proteins

Multiple DNA sequences corresponding to the different isoforms of *NFXL1*, *INSR* and *RYR1* were retrieved from the UCSC genome browser. Next, protein sequences were taken from UniProt, and multiple in-silico tools were used to identify domain structures of the proteins (Supplementary Methods).

### 4.7. INSR Splicing Prediction, Complementary DNA Synthesis and Cloning

The splicing effect of *INSR* variant c.2232+7T>G was also analyzed by the Human Splice Finder (https://hsf.genomnis.com; accessed on 1 March 2024), Berkeley Drosophila Genome Project (https://www.fruitfly.org/seq_tools/splice.html; accessed on 1 March 2024) and SpliceAid (http://www.introni.it/splicing.html; accessed on 1 March 2024) tools. To experimentally determine the effect of the variant, total RNA was extracted using TriZol from a fresh blood sample of patient IV:2 from family PSYAK10. Oligo dT primers (Thermo Fisher Scientific) were used to prepare a cDNA library. *GAPDH* primers were utilized as a positive control (Table S1). A partial *INSR* cDNA sequence (exons 9–13) was amplified by specific primers (Table S1) using Phusion^®^ High-Fidelity DNA polymerase (Thermo Fisher Scientific). The PCR product was cloned into the pJET 1.2/blunt cloning vector (Thermo Fisher Scientific). Competent *E. coli* DH5α cells were transformed with the recombinant vector, and the bacteria were spread on ampicillin, LB agar selection plates. Six positive recombinant plasmids were analyzed using proprietary BT sequencing technology (Celemics, Seoul, South Korea) (Supplementary Methods). The obtained sequences were compared with the cDNA sequence of the wild-type *INSR*.

## 5. Conclusions

This study has increased the genetic spectrum associated with psychiatric illnesses. Functional investigations utilizing animal models or cell lines can delineate the effects of these variants. Such studies will provide insights into the function of the brain in health and disease. Better understanding could pave the way for the development of therapeutic drugs to better treat or cure psychosis.

## Figures and Tables

**Table 1:** Clinical manifestations in the affected individuals of families PSYAK10 and PSYAK22.

Family	Patient	Sex	Current Age (Years)	Age of Onset (Psychosis) (Years)	Age of Death (Years)	Symptoms	Diagnosis	Current Status	Status
PSYAK10	II:2	Male	NA	18	65	Self-smiling, Self-talking, Unable to concentrate, Abusive language, Auditory hallucinations	Schizophrenia	Treatment-resistant	Deceased
	IV:2	Male	54	19	NA	Self-smiling, Self-talking, Aggression, Auditory hallucinations, Catatonia, Spitting on floor, Wandering aimlessly	Schizophrenia	Treatment-resistant	Alive
	IV:3	Male	52	29	NA	Self-smiling, Self-talking, Unable to concentrate, Abusive language, Auditory hallucinations, Spitting on floor, Wandering aimlessly	Schizophrenia	Treatment-resistant	Alive
PSYAK22	IV:1	Female	38	20	NA	Self-Smiling, Self-talking, Irrelevant talk, Auditory hallucinations, Catatonia	Schizophrenia	Stable with medication	Alive
	IV:2	Male	36	23	NA	Self-smiling, Self-talking, Delusions, Unable to concentrate, Auditory and visual hallucinations, Suicidal thoughts	Schizophrenia	Treatment-resistant	Alive

NA = Not applicable or not available.

**Table 2:** Filtered homozygous variants for families PSYAK10 and PSYAK22 after exome data analyses.

Family	Gene	* gDNA Change	RefSeq ID	cDNA and Amino Acid Change	dbSNP	Allele Frequency (%)	Conservation GERP	Predictions						Comments
						gnomAD		SIFT	PolyPhen2	REVEL	MT	FATHMM	SpliceAl	
PSYAK10	*MTF1*	chr1: 38281098 G>C	NM_005955.3	c.1972C>G p.Pro658Ala Missense	*rs534357571*	0.0002006 (no homozygote)	5.03	0.42 B	NA	0.12 B	0.47 B	3.01 U	0.00 B	Predicted mostly benign, amino acid not conserved
	*RLF*	chr1: 40627183 C>T	NM_012421.4	c.112C>T p.Arg38Cys Missense	*rs147792979*	0.0017180 (no homozygote)	2.39	0.008 U	0.12 U	0.06 B	0.99 D	2.33 U	0.00 B	Predicted mostly uncertain or benign, amino acid not conserved
	*CCS*	chr11: 66366958 G>T	NM_005125.2	c.279G>T p.Leu93Leu Synonymous	*rs61731811*	0.0062974 (7 homozygotes)	NA	NA	NA	NA	NA	NA	0.00 B	Not likely to affect splicing, multiple number of homozygotes in controls
	*PC*	chr11: 66617859 G>A	NM_001040716.1	c.2550C>T p.Cys850Cys Synonymous	*rs61749179*	0.0009627 (2 homozygotes)	NA	NA	NA	NA	NA	NA	0.00 B	Not likely to affect splicing
	*ANKRD49*	chr11: 94231641 C>G	NM_017704.3	c.663C>G p.Ile221Met Missense	NA	0	2.78	0.167 B	NA	0.05 B	1 D	−0.52 U	0.00 B	Predicted mostly benign, amino acid not conserved
	*TYRO3*	chr15: 41861163 G>A	NM_006293.4	c.1195G>A p.Ala399Thr Missense	*rs199712738*	0.0001670 (no homozygote)	5.28	0.16 B	0.07 B	0.06 B	0.94 D	0.43 U	0.04 B	Predicted mostly benign, amino acid not conserved
	*MGA*	chr15: 42003006 C>T	NM_001164273.1	c.2543C>T p.Ala848Val Missense	*rs532680103*	0.0001003 (no homozygote)	6.08	0.003 U	0.545 U	0.12 B	0.081 B	2.27 U	0.00 B	Predicted mostly uncertain or benign, amino acid not conserved
	*LCMT2*	chr15: 43621183 C>G	NM_014793.4	c.1505G>C p.Ser502Thr Missense	NA	0	2.34	0.591 B	NA	0.03 B	0.00 B	−0.24 U	0.00 B	Predicted mostly benign, amino acid not conserved
	*LCMT2*	chr15: 43621253 A>G	NM_014793.4	c.1435T>C p.Cys479Arg Missense	*rs1237017176*	0	−2.63	0.23 B	NA	0.09 B	0.630 D	2.49 U	0.00 B	Predicted mostly benign, amino acid not conserved
	*TP53BP1*	chr15: 43784664 C>T	NM_001141980.2	c.10G>A p.Glu4Lys Missense	NA	0	4.28	0 D	NA	0.08 B	1 D	2.6 U	0.03 B	Predicted mostly uncertain or benign, amino acid not conserved
	*CATSPER2*	chr15: 43940173 A>G	NM_172095.3	c.87T>C p.Ile29Ile Synonymous	*rs28494549*	0.0004175 (2 homozygotes)	NA	NA	NA	NA	NA	NA	0.00 B	Not likely to affect splicing
	*PDIA2*	chr16: 334503 C>T	NM_006849.3	c.316C>T p.Arg106Cys Missense	*rs199711437*	0.0000248 (no homozygote)	−0.905	0.023 U	0.118 U	0.15 B	0 B	1.02 U	0.00 B	Predicted mostly uncertain or benign, amino acid not conserved
	*CACNA1H*	chr16: 1250451 A>G	NM_021098.3	c.999A>G p.Ala333Ala Synonymous	*rs529471626*	0.0005692 (2 homozygotes)	NA	NA	NA	NA	NA	NA	0.00B	Not likely to affect splicing
	*TSC2*	chr16: 2122977 C>G	NM_000548.5	c.2348 C>G p.Thr783Ser Missense	*rs562945619*	0.0008334 (4 homozygotes)	3.59	0.63 B	0.01 B	0.24 B	0.00027 B	−2.13 U	0.00 B	Predicted mostly benign, amino acid not conserved, multiple number of homozygotes in controls
	*ANKS3*	chr16: 4747407 G>A	NM_133450.3	c.1821C>T p.Gly607Gly Synonymous	*rs146041043*	0.0033007 (4 homozygotes)	NA	NA	NA	NA	NA	NA	0.00 B	Not likely to affect splicing, multiple number of homozygotes in controls
	*SHD*	chr19: 4280266 C>A	NM_020209.3	c.206C>A p.Ala69Asp Missense	*rs535190570*	0.0004124 (1 homozygote)	2.36	0.20 B	0.11 B	0.06 B	0.007 B	0.92 U	0.04 B	Predicted mostly benign, amino acid not conserved
	*CHAF1A*	chr19: 4432011 G>A	NM_005483.3	c.2010G>A p.Glu670Glu Synonymous	*rs11556317*	0.0023824 (3 homozygotes)	NA	NA	NA	NA	NA	NA	0.00 B	Not likely to affect splicing
	*CATSPERD*	chr19: 5778486 C>G	NM_152784.4	c.2196C>G p.Ser732Arg Missense	NA	0	1.19	0.037 U	NA	0.08 B	0 B	1.91 U	0.03 B	Predicted mostly uncertain or benign, amino acid not conserved
	*PRR22*	chr19: 5783074 G>A	NM_001134316.2	c.1184C>T p.Pro395Leu Missense	*rs199650444*	0.0000575 (no homozygote)	1.26	0.38 B	NA	0.01 B	0 B	0.92 U	0.00 B	Predicted mostly benign, amino acid not conserved
	***INSR***	**chr19:** **7150550** **A>C**	**NM_000208.4**	**c.2232-7** **T>G** Splicing **	***rs775596300***	**0.0000717** **(no homozygote)**	**NA**	**NA**	**NA**	**NA**	**NA**	**NA**	**0.65** **D**	**Selected, as variant was predicted to affect splicing**
	*STXBP2*	chr19: 7712143 C>T	NM_006949.4	c.1538+10C>T Splicing	*rs139200597*	0.0017607 (3 homozygotes)	NA	NA	NA	NA	NA	NA	0.00 B	Predicted mostly uncertain or benign, intronic variant, nucleotide not conserved
	*ZNF493*	chr19: 21606806 A>G	NM_001076678.2	c.1345A>G p.Thr449Ala Missense	*rs553037933*	0.0006879 (1 homozygote)	−0.131	0.178 B	0.084 B	0.02 B	0 B	1.31 U	0.00 B	Predicted mostly uncertain or benign, amino acid not conserved
PSYAK22	*NOTCH2*	chr1: 120479948 T>C	NM_024408.4	c.3479A>G p.His1160Arg Missense	*rs142876168*	0.001268652 (2 homozygotes)	5.06	0.002 U	0.42 U	0.632 U	1 D	−2.44 U	0.00 B	Predicted mostly uncertain or benign, amino acid not conserved
	*WDR19*	chr4: 39271606 G>A	NM_025132.4	c.3369G>A p.Arg1123Arg Synonymous	*rs775035034*	0.0000240944 (no homozygote)	NA	NA	NA	NA	NA	NA	0.00 B	Not likely to affect splicing
	*N4BP2*	chr4: 40104739 C>A	NM_018177.6	c.1274C>A p.Thr425Asn Missense	*rs62621880*	0.001714672 (3 homozygotes)	4.34	0.02 U	0.52 U	0.06 B	0.0005 B	2.26 U	0.00 B	Predicted mostly uncertain or benign, amino acid not conserved
	***NFXL1***	**chr4:** **47898547** **C>T**	**NM_001278624.2**	**c.1322G>A** **p.Cys441Tyr** **Missense**	***rs748118226***	**0.000003985** **(no homozygote)**	**4.69**	**0** **D**	**1** **D**	**0.74** **D**	**1** **D**	**−0.18** **U**	**0.00** **B**	**Selected due to high damaging prediction scores, amino acid was conserved**
	*NFXL1*	chr4: 47900044 C>T	NM_001278624.2	c.1144G>A p.Val382Ile Missense	*rs751474556*	0.00006793640 (1 homozygote)	2.83	0.204 B	0.005 B	0.063 B	0.007 B	0.99 U	0.00 B	Predicted mostly benign, amino acid not conserved
	*CNGA1*	chr4: 47938559 A>G	NM_001379270.1	c.1940T>C p.Met647Thr Missense	*rs776545639*	0.000208562 (1 homozygote)	3.57	0.808 B	0.003 B	0.291 B	0.02 B	−4.04 U	0.00 B	Predicted mostly benign, amino acid not conserved
	*ADGRL3*	chr4: 62598944 A>G	NM_001387552.1	c.1071A>G p.Gln357Gln Synonymous	*rs530970218*	0.000947856 (no homozygote)	NA	NA	NA	NA	NA	NA	0.00 B	Not likely to affect splicing
	*KIAA1549*	chr7: 138601730 G>A	NM_001164665.2	c.2642C>T p.Thr881Met Missense	*rs138709216*	0.000425196 (1 homozygote)	4.39	0.13 B	0.11 B	0.06 B	0 B	1.78 U	0.00 B	Predicted mostly benign, amino acid not conserved
	*KIAA1549*	chr7: 138602970 C>T	NM_001164665.2	c.1402G>A p.Val468Ile Missense	*rs545645203*	0.000100431 (no homozygote)	−7	1 B	0.004 B	0.003 B	0 N	1.97 U	0.00 B	Predicted mostly benign, amino acid not conserved
	*EPHA1*	chr7: 143094434 C>T	NM_005232.5	c.1734G>A p.Gln578Gln Synonymous	*rs542197026*	0.001268619 (no homozygote)	NA	NA	NA	NA	NA	NA	0.00 B	Not likely to affect splicing
	*ITPR2*	chr12: 26784841 C>T	NM_002223.4	c.2892G>A p.Val964Val Synonymous	*rs199942805*	0.000910506 (no homozygote)	NA	NA	NA	NA	NA	NA	0.00 B	Not likely to affect splicing
	*ZXDA*	chrX: 57936451-57936453 delinsGGC	NM_007156.5	c.402_404delinsGCC p.Cys135Pro Missense	*rs758668472* and *rs753063356*	0.000356035 (1 homozygote)	NA	NA	NA	NA	NA	NA	0.00 B	Very common with a frequency of >10% in in-house data of 300 individuals, including multiple homozygous genotypes

* Variant positions according to the Human Feb. 2009 (GRCh37/hg19) Assembly. RefSeq = Reference Sequence, gnomAD = Genome Aggregation Database (accessed on 1 March 2024), GERP = Genomic Evolutionary Rate Prediction (negative and low scores indicate no or low conservation), SIFT = Sorting Intolerant from Tolerant algorithm, PolyPhen2 = Polymorphism phenotyping v2, REVEL = Rare Exome Variant Ensemble Learner, MT = Mutation Taster, FATHMM = Functional Annotation Through Hidden Markov Models, SpliceAI = Splice-Altering algorithm, D = Damaging or Deleterious, T = Tolerated, NA = Not applicable, B = Benign, U = Uncertain. ** Splicing predictions were also completed using Human Splice Finder (https://hsf.genomnis.com; accessed on 1 March 2024) and other software, which predicted a deleterious effect as well.

## Data Availability

All the data are presented in the manuscript and the Supplementary Files or can be obtained from the corresponding authors on reasonable request.

## Supplementary Materials

The supporting information can be downloaded at https://www.mdpi.com/article/10.3390/ijms26104925/s1.
