# Supplementary material for: Rare Homozygous Variants in INSR and NFXL1 Are Associated with Severe Treatment-Resistant Psychosis
Source: Int J Mol Sci. 2025 May 21;26(10):4925. doi: 10.3390/ijms26104925 (PMC12111829; doi:10.3390/ijms26104925)
Supplement: Supplementary file 1 [file ijms-26-04925-s001.zip › ijms-3618587-supplementary.pdf]

Supplementary Methods

Clinical Assessments

Detailed clinical assessments were performed for all participants by a psychiatrist (SS) and a psychologist (AI) using Diagnostic Interview for Genetic Studies (DIGS) [1] with modified MiniMental Status Examination (mMMSE) [2], Hamilton Depression and Anxiety Rating Scales (HAM-D, HAM-A) [3], Diagnostic Interview for Psychosis and Affective Disorders (DI-PAD) [4], and Positive and Negative Syndrome Scale (PANSS) [5]. These tools confirmed the presence of psychoses in the affected individuals of families and the absence of psychiatric symptoms in any of the unaffected relatives. Patients were free of confounding diagnoses such as other psychiatric disorders or intellectual disabilities. Parental consanguinity, family history, kinship and medication use were ascertained through questioning from the legal guardians. Medical records of the patients maintained at the hospital were also accessed. For affected individuals of family PSYAK10, initial diagnosis and detailed clinical histories were ascertained from multiple psychiatrists at the hospital, since the patients had been hospitalized continuously for at least previous twenty years with minimum contact with their families. First degree relatives were contacted later. Written informed consents were taken from the participants or the legal guardians.

Sampling and DNA analyses

Blood samples of the patients were collected from the hospital, whereas the first degree relatives were visited at their homes. DNA extraction from 5-10 ml whole-blood was performed using sucrose lysis and salting out. Agilent SureSelect V7-postcapture kit (Agilent Technologies, Santa Clara, CA, USA) was used for exome sequencing of the DNA samples on an Illumina NovaSeq6000 sequencer (Macrogen Inc, Seoul, South Korea). To select exonic and splice-site variants with allele frequencies less than 1%, different public databases were used including GenomeAsia (https://www.genomeasia100k.org/), NHLBI GO Exome Sequencing Project 6500 (ESP6500; https://esp.gs.washington.edu/drupal/), gnomAD (https://gnomad.broadinstitute.org/), GME Variome (https://illumina.github.io/NirvanaDocumentation/data-sources/gme/), 1000 Genomes (https://www.internationalgenome.org/), Iranome (http://www.iranome.ir/), ExAC (http://exac.broadinstitute.org) and TOPMeD Bravo (https://bravo.sph.umich.edu/). Multiple

pathogenicity prediction tools were used including FATHMM (http://fathmm.biocompute.org.uk/), REVEL (https://sites.google.com/site/revelgenomics/), SIFT (http://sift-dna.org), MT (https://www.mutationtaster.org/), PolyPhen2 (http://genetics.bwh.harvard.edu/pph2/) and SpliceAI (https://spliceailookup.broadinstitute.org/). Conservation of the amino acid affected by the variants was assessed using GERP scores (http://mendel.stanford.edu/sidowlab/downloads/gerp/index.html) as well as by accessing alignments of the orthologues from the UCSC genome browser (https://genome.ucsc.edu/) and HomoloGene (https://www.ncbi.nlm.nih.gov/homologene).

Homozygosity analyses

Visual representation of each chromosome was obtained using AgileMultideogram software (https://dna-leeds.co.uk/agile/) which depicted autozygous/homozygous regions in yellow, red or blue colors, depending on whether they were identified in patients, common in affected individuals or present in unaffected individuals. Subsequently, shared regions of homozygosities (ROH) were further scrutinized using AgileVCFMapper (http://www.dna-leeds.co.uk/agile/AgileVCFMapper/) with standard settings[6] for a closer examination of individuals' chromosomal regions.

Allele-specific PCR

Competitive allele-specific PCR was performed in two separate reactions for the mutant and the wild-type alleles of *INSR*, so that each reaction had an allele specific and respective forward and reverse primers for amplifications (three primers in one reaction). Tetra primer ARMS PCR was completed separately for the *NFXL1* and the two *RYR1* variants. For each reaction for detection of both the wild-type and variant alleles for *NFXL1* or *RYR1*, four respective primers (two allele specific and respective forward and reverse primers) were used in one reaction. At least 102 ethnically matched controls were genotyped for each variant. The segregation of *RYR1* variants in members of family PSYAK8 was also completed using Tetra primer ARMS PCR.

Protein modeling and domain analyses studies

The stabilities of both the wild-type and the mutated proteins due to the missense variants were assessed by employing online tools such as ExPASy ProtParam (https://web.expasy.org/protparam/) and I-Mutant Suite (http://gpcr2.biocomp.unibo.it/cgi/predictors/I-Mutant3.0/I-Mutant3.0.cgi). Several *in silico* online tools, including CUPSAT (http://cupsat.tu-bs.de), PredictProtein (https://predictprotein.org/); SNAP2 (https://rostlab.org/services/ snap/); and HOPE tool (https:// www3.cmbi.umcn.nl/hope) were utilized to examine the projected impacts of the variants on the proteins' localization and conformation. Structural illustrations were prepared using the PyMoL program (https://pymol.org/2/) for the wild-type protein sequences.

The protein sequences of INSR, NFXL1 and RYR1 were obtained from UniProt (https://www.uniprot.org/). Multiple *in silico* tools including ScanProsite-ExPasy (https://prosite.expasy.org/scanprosite/); Pfam (http://pfam.xfam.org/); InterPro (https://www.ebi.ac.uk/interpro/); and SMART-EMBL (https://smart.embl.de/) were used to identify the domain structures of the proteins.

BTseq procedure

Btseq (Celemics, Seoul, South Korea) was performed for the recombinant plasmids on an Illumina MiSeq instrument. This involved the preparation of barcoded libraries from the plasmids and massively parallel sequencing to yield the final sequences. The final data were provided as .ab1files which were examined using SeqMan software (DNAStar, Lasergene).

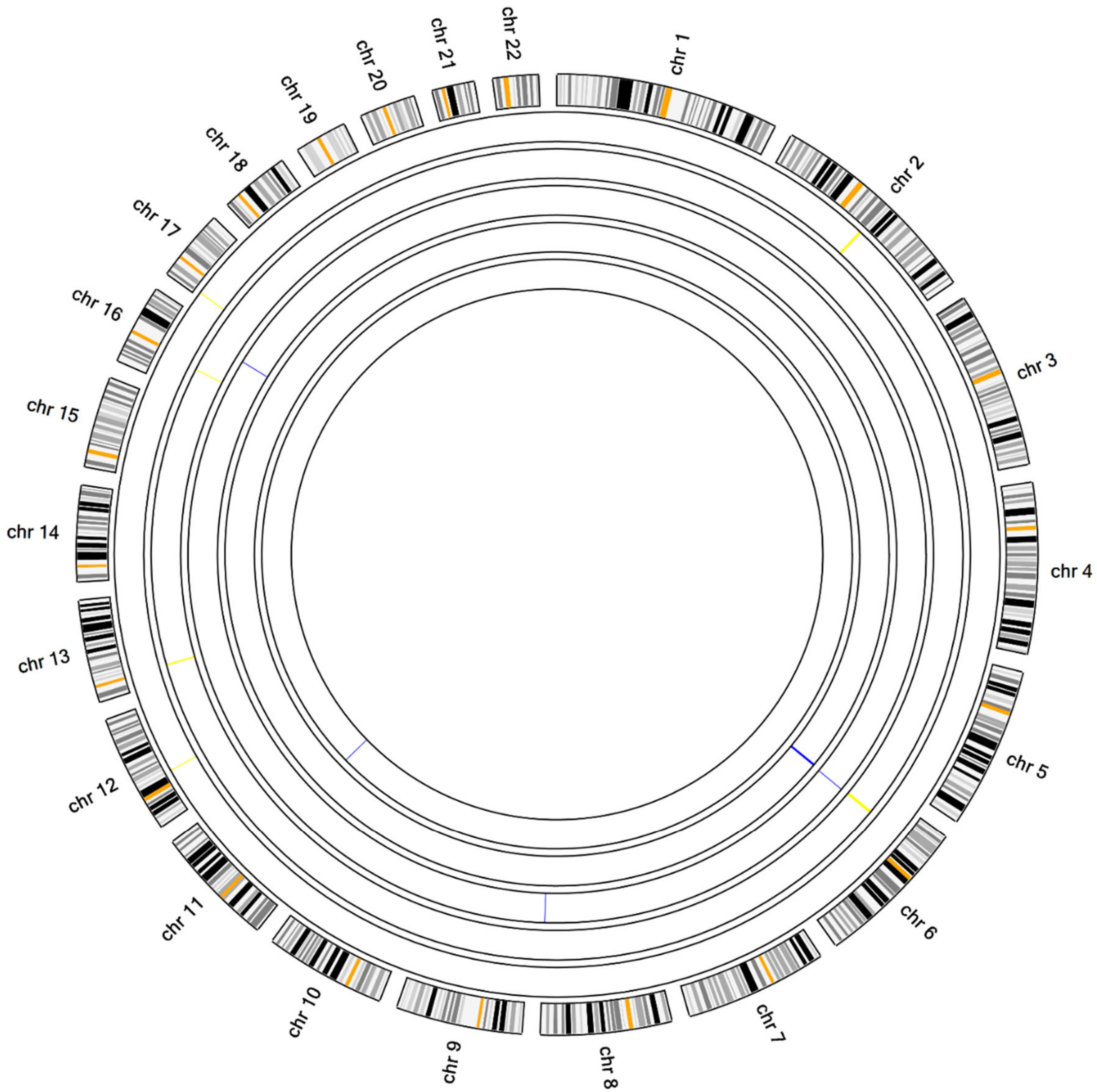
chr 1
chr 2
chr 3
chr 4
chr 5
chr 6
chr 7
chr 8
chr 9
chr 10
chr 11
chr 12
chr 13
chr 14
chr 15
chr 16
chr 17
chr 18
chr 19
chr 20
chr 21
chr 22

Supplementary Figure 1: Representation of results after autozygosity mapping of exome data of all members of family PSYAK1. Autozygous/homozygous regions were identified in exome variant data of the five participants and plotted using AgileMultideogram software (https://dna-leeds.co.uk/agile/) for chromosomes 1-22. The outer most rectangular bands arranged in a circular form, represent all the chromosomes (labeled) and their cytological banding patterns. Next, exome variant data of all chromosomes for individuals VI:2, VI:3, IV:4, VI:1 and V:2 from pedigree PSYAK1 are shown as white double circular bands from the outer to inner circles, respectively. Individuals VI:2 and VI:3 are the patients. The yellow color indicates the region of homozygosity detected for each affected individual, while the blue color shows the homozygous regions in any of the three unaffected individuals' data. Comparison of this data clearly indicates that though all individuals do have multiple autozygous/homozygous regions spread on different chromosomes, the two patients do not have any one region of homozygosity in common.

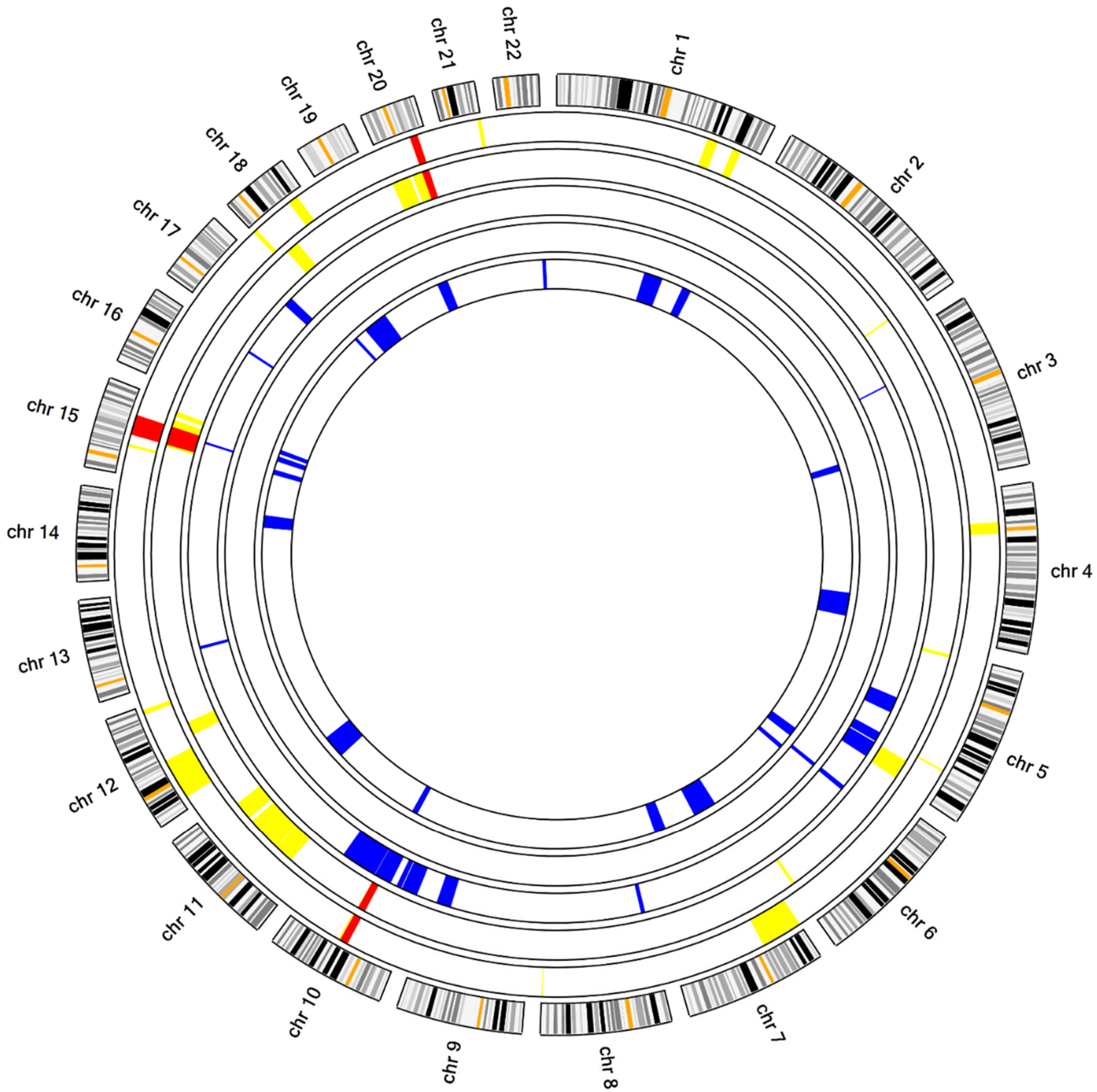
chr 1
chr 2
chr 3
chr 4
chr 5
chr 6
chr 7
chr 8
chr 9
chr 10
chr 11
chr 12
chr 13
chr 14
chr 15
chr 16
chr 17
chr 18
chr 19
chr 20
chr 21
chr 22

Supplementary Figure 2: Autozygosity mapping of exome data of all members of family PSYAK4. Autozygous/homozygous regions were identified in exome variant data of the five participants and plotted using AgileMultideogram software (https://dna-leeds.co.uk/agile/) for chromosomes 1-22. The outer most rectangular bands arranged in a circular form, represent all the chromosomes (labeled) and their cytological banding patterns. Next, exome variant data of all chromosomes for individuals IV:2, IV:3, III:2, III:3 and IV:1 from pedigree PSYAK4 are shown as white double circular bands from the outer to inner circles, respectively. Individuals IV:2 and IV:3 are the patients. The yellow color indicates the regions of homozygosities detected for each affected individual, red stretches represent regions of autozygosity/homozygosity common in two affected individuals suffering from schizophrenia while the blue color shows the homozygous regions in any of the three unaffected individuals' data. Comparison of this data indicates the presence of three regions of homozygosities (ROH) on chromosome 10q, 15q and 20q common to both patients IV:2 and IV:3 but none of the deleterious variant were found in these ROH (Supplementary table 2).

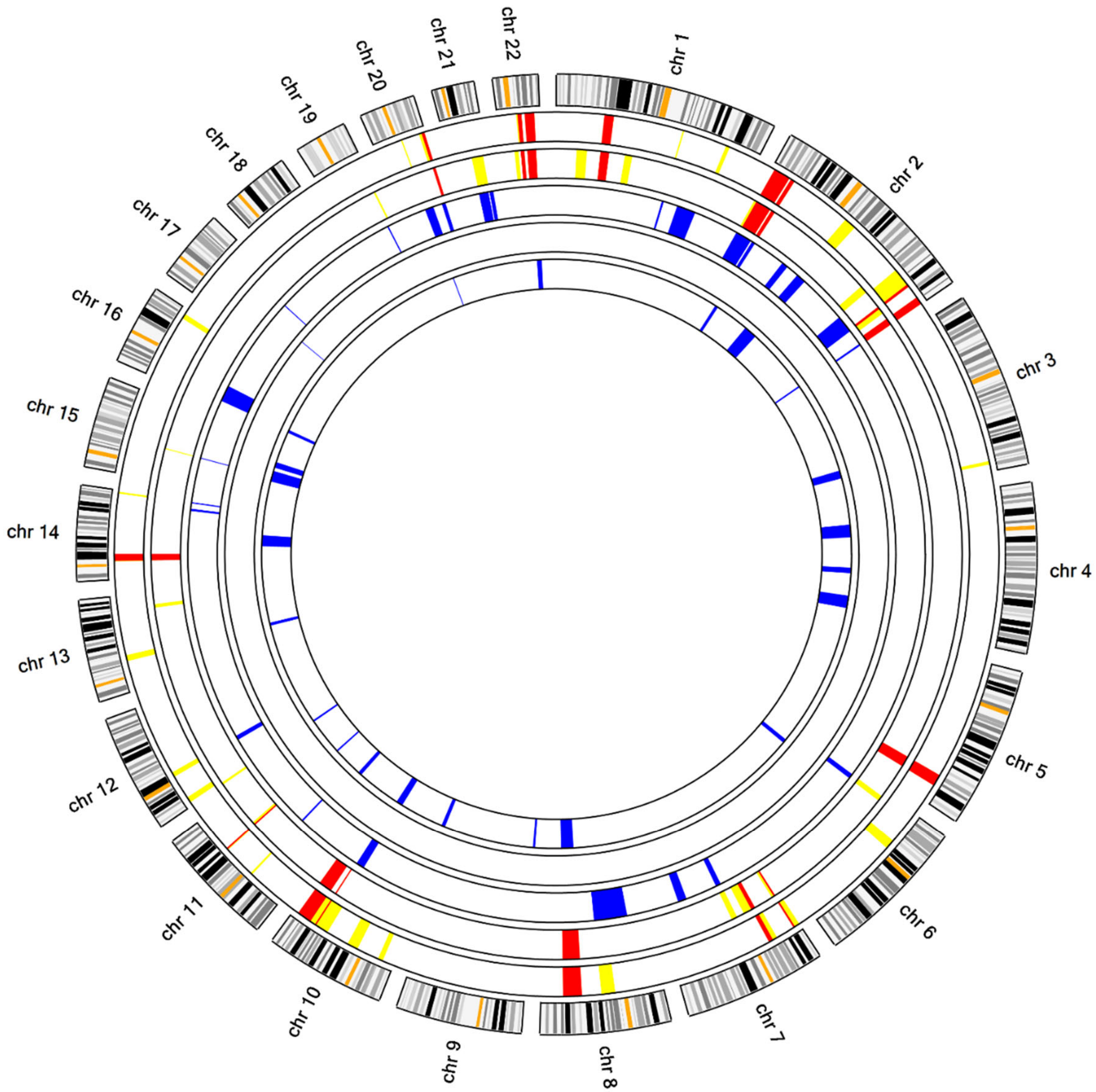
chr 1
chr 2
chr 3
chr 4
chr 5
chr 6
chr 7
chr 8
chr 9
chr 10
chr 11
chr 12
chr 13
chr 14
chr 15
chr 16
chr 17
chr 18
chr 19
chr 20
chr 21
chr 22

Supplementary Figure 3: Autozygosity mapping of exome data of all members of family PSYAK5 in which one sister IV:2 and one brother IV:3 were affected with bipolar disorder accompanied by psychosis [7]. Exome variant data of the five participants was plotted using AgileMultideogram software (https://dna-leeds.co.uk/agile/) for chromosomes 1-22. The rectangular bands encircling the outermost layer in a circular fashion symbolize the entirety of chromosomes with their distinctive cytological banding patterns. Next, exome variant data of all chromosomes for individuals IV:2, IV:3, IV:4, III:2 and III:3 from pedigree PSYAK5 are shown as white double circular bands from the outer to inner circles, respectively. Individuals IV:2 and IV:3 are the patients. The yellow color indicates the regions of homozygosities detected for each affected individual, red stretches represent regions of autozygosity/homozygosity common to two affected individuals suffering from psychotic bipolar disorder while the blue color shows the homozygous regions in any of the three unaffected individuals' data. Comparison of this data indicates the presence of multiple regions of homozygosities (ROH) on chromosomes 1p, 2p, 2q, 5q, 8q, 10q, 14q, 20q and 22q with two very small ROH on 7p and 11q shared by two patients IV:2 and IV:3. However, none of the variants in these ROH were found deleterious (Supplementary table 2).

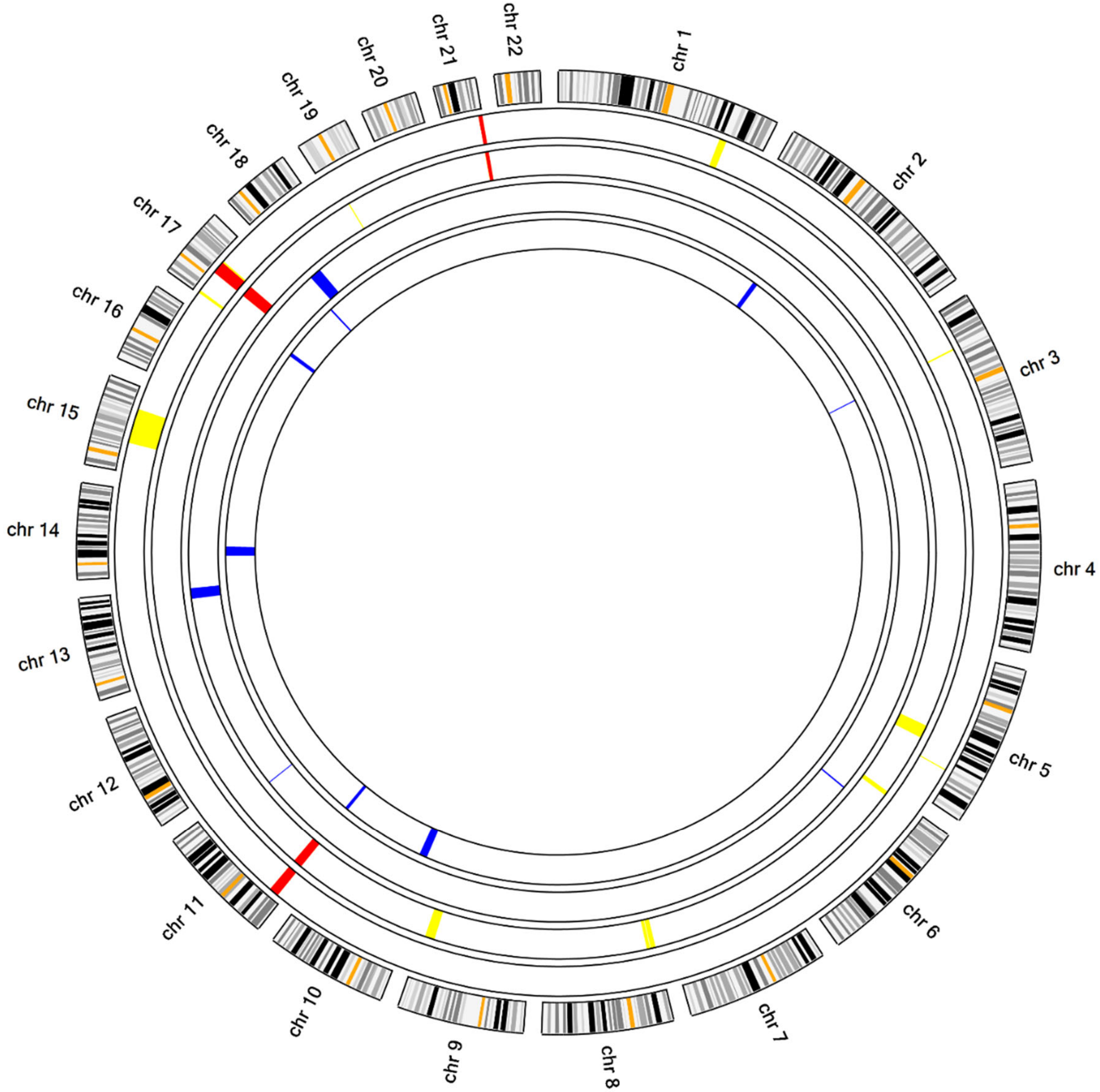
chr 1
chr 2
chr 3
chr 4
chr 5
chr 6
chr 7
chr 8
chr 9
chr 10
chr 11
chr 12
chr 13
chr 14
chr 15
chr 16
chr 17
chr 18
chr 19
chr 20
chr 21
chr 22

Supplementary Figure 4: Illustration of autozygosity mapping using exome data from all members of family PSYAK6, where two sisters VI:1 and VI:2 were affected by schizophrenia [7]. The AgileMultideogram software (https://dna-leeds.co.uk/agile/) was utilized to visualize the exome variant data of the four participants across chromosomes 1-22. Circular bands surrounding the outer layer represent the chromosomes with their unique cytological banding patterns. Subsequently, the exome variant data for individuals VI:1, VI:2, V:2, and VI:3 from pedigree PSYAK6 [7], respectively is displayed as white double circular bands, transitioning from outer to inner circles. Yellow highlights signify regions of homozygosity identified in each affected individual, while red stretches denote regions of autozygosity/homozygosity shared by the two affected individuals and blue color represents homozygous regions in any of the two unaffected individuals' data. Examination of this data reveals the presence of multiple regions of homozygosities (ROH) on chromosomes 11p and 17q, with a very small ROH on chromosome 21q. No deleterious variants were found within these regions (Supplementary table 2).

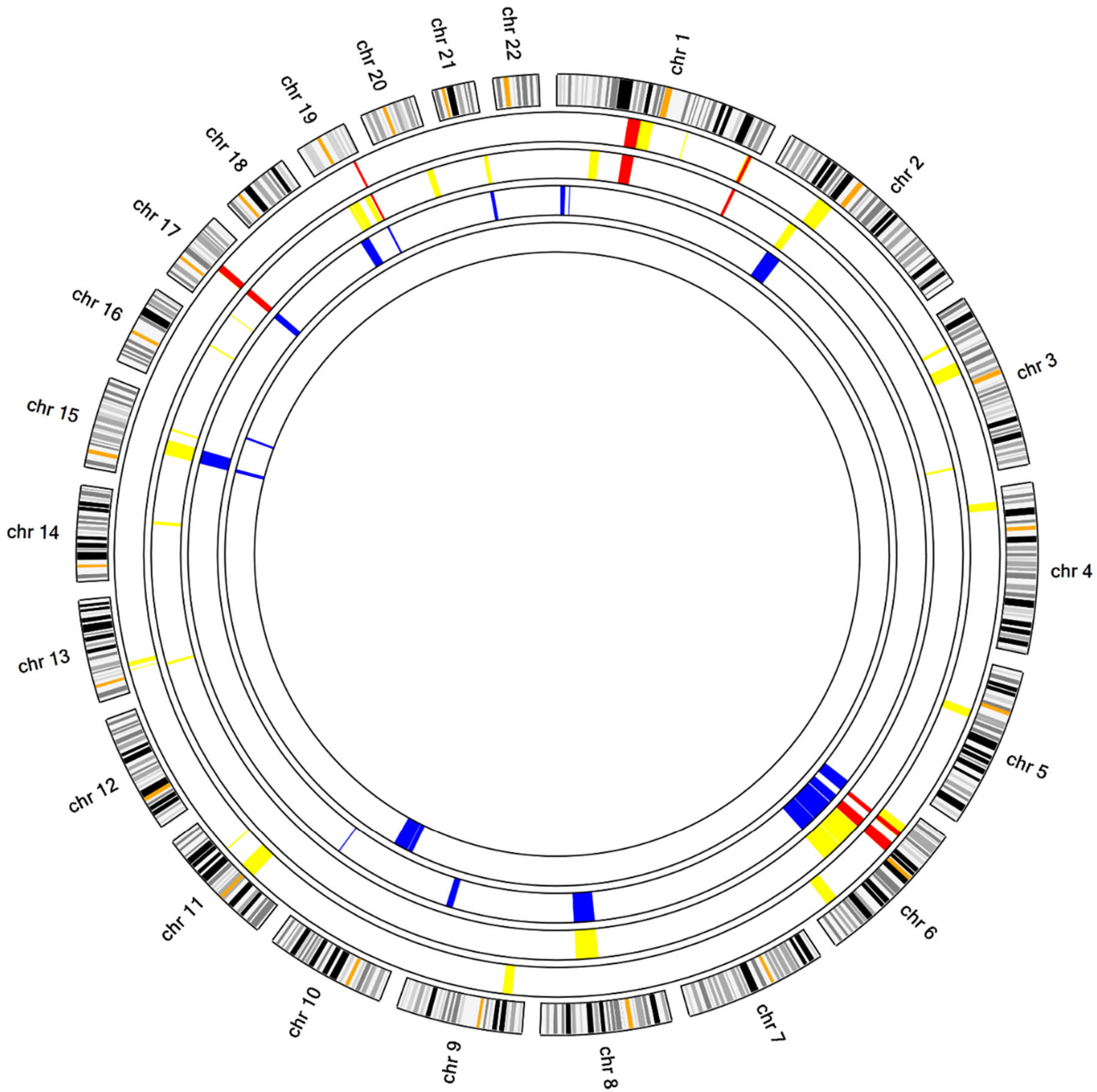
chr 1
chr 2
chr 3
chr 4
chr 5
chr 6
chr 7
chr 8
chr 9
chr 10
chr 11
chr 12
chr 13
chr 14
chr 15
chr 16
chr 17
chr 18
chr 19
chr 20
chr 21
chr 22

Supplementary Figure 5: Autozygosity mapping of exome data of all members of family PSYAK7 in which a brother IV:2 and his sister IV:3 were affected with treatment-resistant schizophrenia. Exome variant data of the four participants was plotted using AgileMultideogram software (https://dna-leeds.co.uk/agile/) for chromosomes 1-22. The rectangular bands encircling the outermost layer in a circular fashion symbolize the entirety of chromosomes with their distinctive cytological banding patterns. Next, exome variant data of all chromosomes for individuals IV:2, IV:3, IV:1 and III:2 from pedigree PSYAK7 are shown as white double circular bands from the outer to inner circles, respectively. The yellow color indicates the regions of homozygosities detected for each affected individual, red stretches represent regions of autozygosity/homozygosity common to both affected individuals suffering from schizophrenia while the blue color shows the homozygous regions in any of the three unaffected individuals' data. Comparison of this data indicates the presence of multiple regions of homozygosities (ROH) on chromosomes 1p and 6p with small ROH on 1q, 6p, 17q and 19q but none of the variants in these ROH were found deleterious (Supplementary table 2).

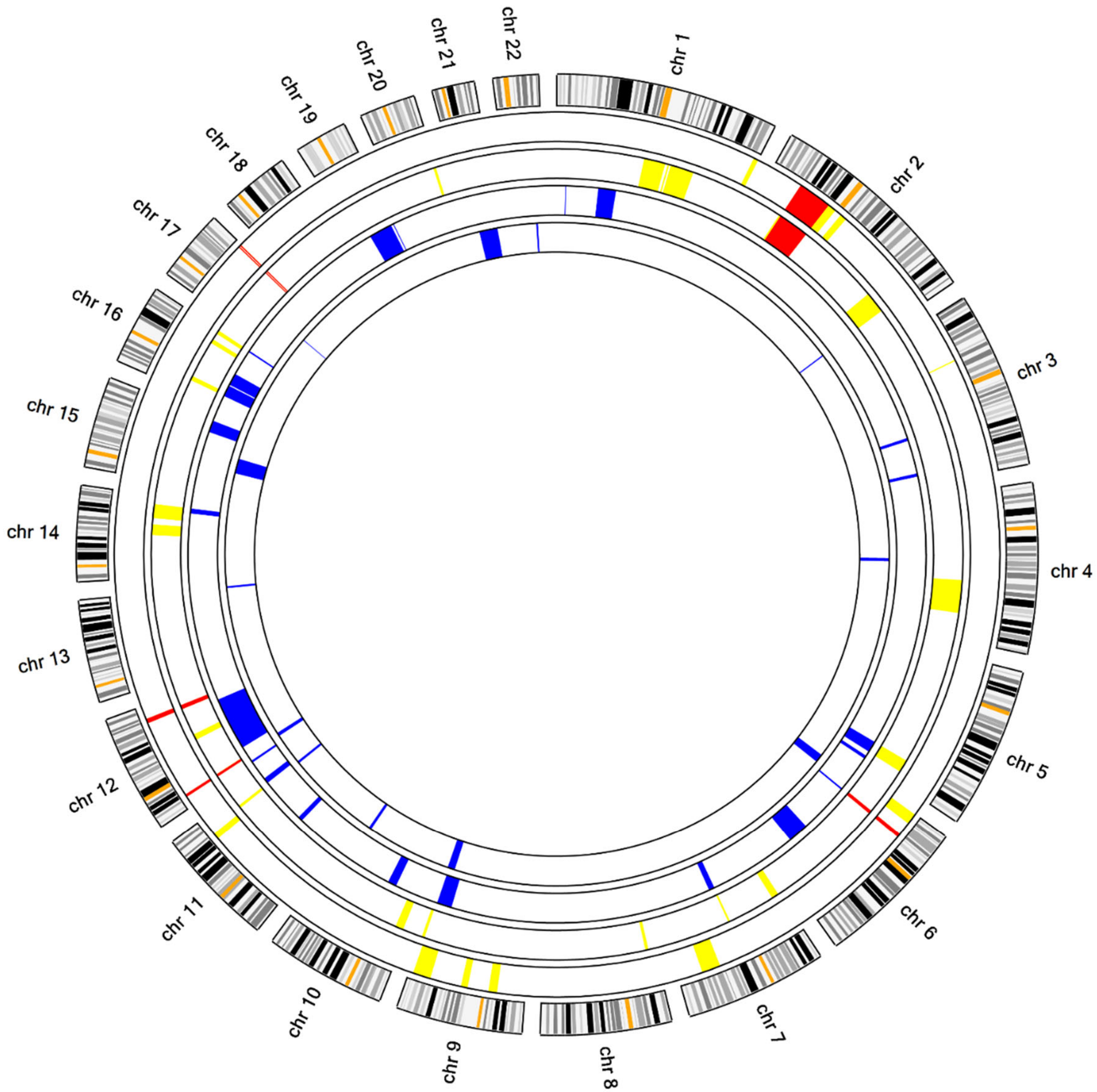
chr 1
chr 2
chr 3
chr 4
chr 5
chr 6
chr 7
chr 8
chr 9
chr 10
chr 11
chr 12
chr 13
chr 14
chr 15
chr 16
chr 17
chr 18
chr 19
chr 20
chr 21
chr 22

Supplementary Figure 6: Autozygosity mapping of exome data for all members of the family PSYAK8, wherein two individuals IV:2 and IV:3 were affected with bipolar disorder accompanied by psychosis. Exome variant data of the four participants was visually represented across chromosomes 1-22. The circular bands enveloping the outermost layer in a radial manner symbolically represent the complete set of chromosomes along with their unique cytological banding patterns. Subsequently, the exome variant information for affected individuals IV:2, IV:3, unaffected sibling IV:1 and their mother III:3 from the PSYAK8 pedigree is illustrated as white dual circular bands progressing from the outermost to inner circles, respectively. The yellow color delineates the regions of homozygosities identified in each affected individual, while the red segments depict regions of autozygosity/homozygosity shared by two affected individuals suffering from psychotic bipolar disorder. In contrast, the blue color highlights the homozygous regions present in the data of any of the two unaffected individuals. Analysis of this dataset reveals the existence of a large region of homozygosity (ROH) on chromosome 2p with multiple small ROH on chromosomes 6p, 12p, 12q and 17q. However, none of the variants within these ROH were predicted to be deleterious (Supplementary table 2).

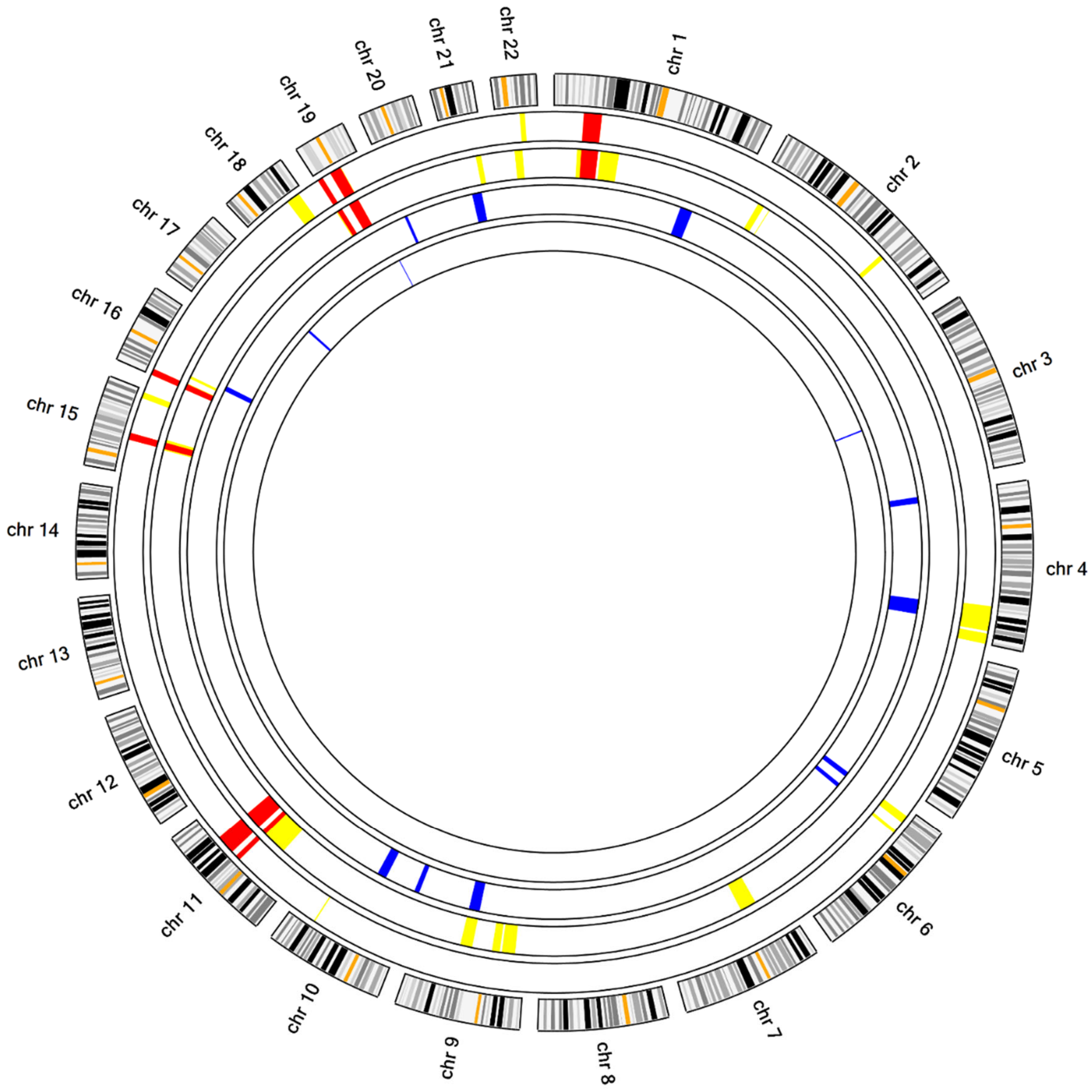
chr 1
chr 2
chr 3
chr 4
chr 5
chr 6
chr 7
chr 8
chr 9
chr 10
chr 11
chr 12
chr 13
chr 14
chr 15
chr 16
chr 17
chr 18
chr 19
chr 20
chr 21
chr 22

Supplementary Figure 7: Autozygosity mapping of exome data of all members of family PSYAK10 in which two brothers IV:2 and IV:3 were affected with treatment-resistant schizophrenia. Exome variant data of the four participants was plotted using AgileMultideogram software (https://dna-leeds.co.uk/agile/) for chromosomes 1-22. The rectangular bands encircling the outermost layer in a circular fashion symbolize the entirety of chromosomes with their distinctive cytological banding patterns. Next, exome variant data of all chromosomes for individuals IV:2, IV:3, IV:1 and III:3 are shown as white double circular bands from the outer to inner circles, respectively. The yellow color indicates the regions of homozygosities detected for each affected individual, red stretches represent regions of autozygosity/homozygosity common in two affected individuals suffering from schizophrenia while the blue color shows the homozygous regions in any of the three unaffected individuals' data. Comparison of this data indicates the presence of multiple regions of homozygosities (ROH) on chromosomes 1p, 11q and 19p with very small ROH on 11q, 15q, 16p and 19p. Variant of interest *INSR* c.2232-7 T>G (Table 2) was found within the large ROH on chromosome 19p.

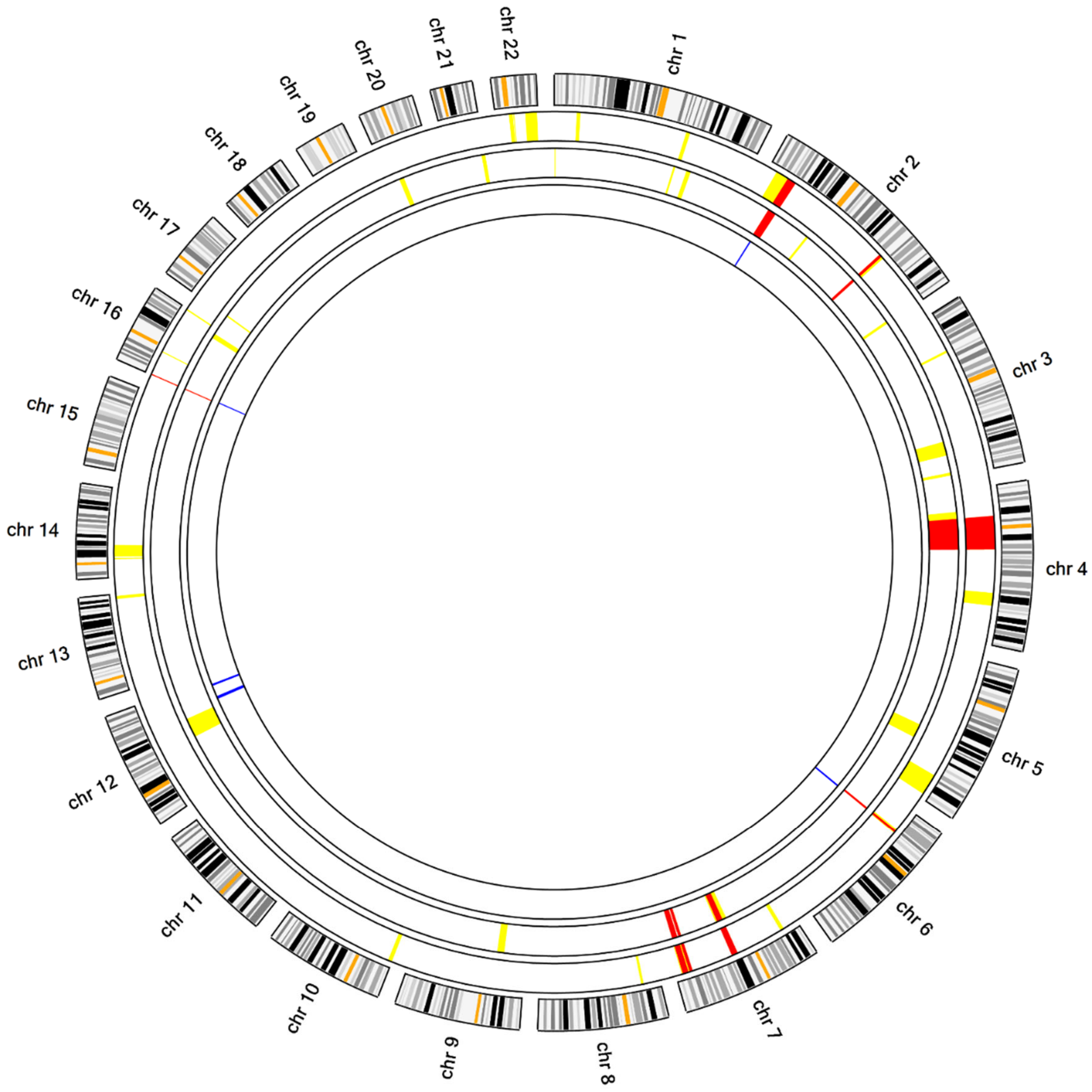
chr 1
chr 2
chr 3
chr 4
chr 5
chr 6
chr 7
chr 8
chr 9
chr 10
chr 11
chr 12
chr 13
chr 14
chr 15
chr 16
chr 17
chr 18
chr 19
chr 20
chr 21
chr 22

Supplementary Figure 8: Illustration of autozygosity mapping derived from the exome data of family PSYAK22, where a sister IV:1 and her brother IV:2 exhibited symptoms of severe treatment-resistant schizophrenia. Utilizing AgileMultideogram software (https://dna-leeds.co.uk/agile/), the exome variant data of three participants IV:1, IV:2 and their father III:2 was visually represented for chromosomes 1-22. The outermost rectangular bands in a circular arrangement symbolically portray the complete set of autosomal chromosomes with their unique cytological banding patterns. Following this, the exome variant data for individuals IV:1, IV:2 and III:2 from pedigree PSYAK22 is depicted as white double circular bands progressing from the outer to inner circles, respectively. The yellow color highlights regions of homozygosity specific to each affected individual, while red segments denote regions of autozygosity/homozygosity shared by the two affected individuals diagnosed with schizophrenia. Blue shade indicates homozygous regions within the data of any of the unaffected individual. Analysis of this data reveals the existence of numerous regions of homozygosity on chromosomes 4p, 4q and 7q, along with minimal common regions of homozygosities on 2q and 7q. The variant *NFXL1* c.1322G>A; p.(Cys441Tyr) located within ROH on chromosome 4 was predicted to be detrimental (Table 2).

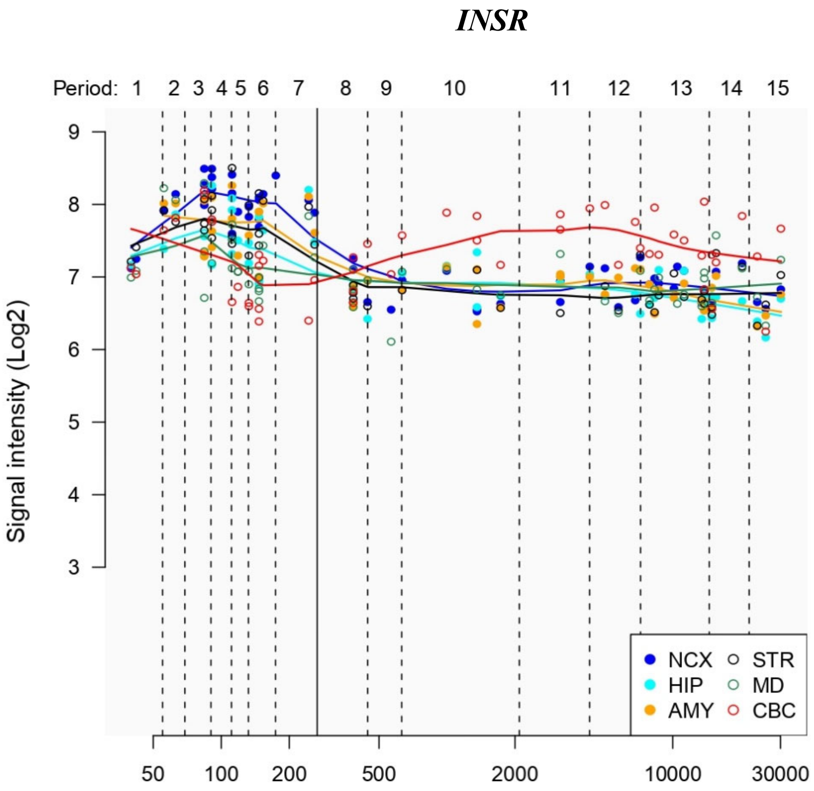

Supplementary Figure 9: Transcriptome analysis of the human brain (https://hbatlas.org/pages/hbtd) reveals the presence of dynamic gene expression of *INSR* throughout the course of development and into adulthood across various brain regions. These regions include the mediodorsal nucleus of the thalamus (MD), the hippocampus (HIP), the cerebellar cortex (CBC), the striatum (STR), the amygdale (AMY), and 11 other areas of the neocortex (NCX). *INSR* has the highest expression in the neocortex and cerebral cortex regions of the human brain.

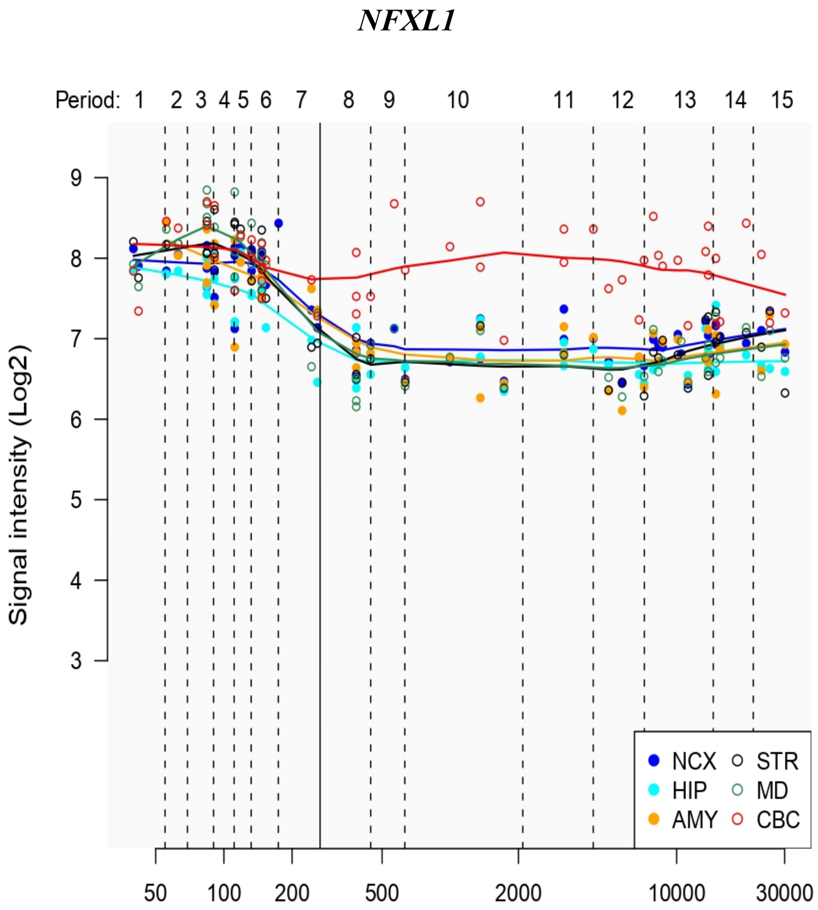

Supplementary Figure 10: Human brain transcriptome analysis (https://hbatlas.org/pages/hbtd) reveals the presence of a dynamic gene expression of *NFXL1* throughout the course of development and into adulthood in various regions of the brain. These regions include the mediodorsal nucleus of the thalamus (MD), the hippocampus (HIP), the cerebellar cortex (CBC), the striatum (STR), the amygdala (AMY), as well as 11 other areas of the neocortex collectively referred to as NCX. The expression of *NFXL1* was found to be highest in the mediodorsal nucleus of the thalamus and the cerebral cortex regions of the human brain.

RYR1

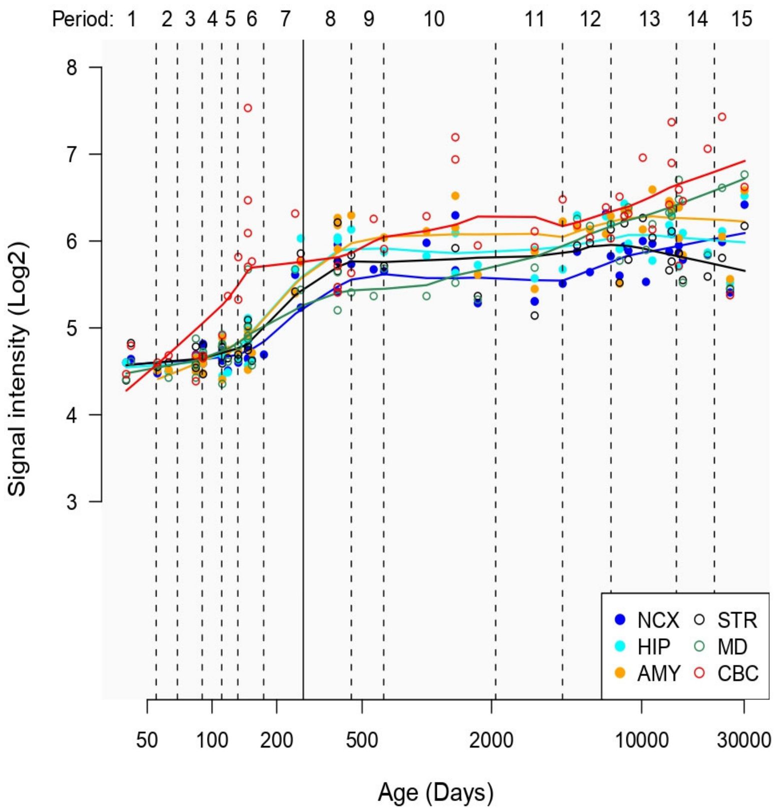

Supplementary Figure 11: Human brain transcriptome analysis indicates dynamic gene expression of *RYR1* during development and adulthood in different regions of the brain (https://hbatlas.org/pages/hbtd). MD, mediodorsal nucleus of the thalamus, HIP, hippocampus, CBC, cerebellar cortex, STR, striatum, AMY, amygdale, NCX, 11 other areas of the neocortex. *RYR1* has the highest expression in the cerebral cortex region of the human brain.

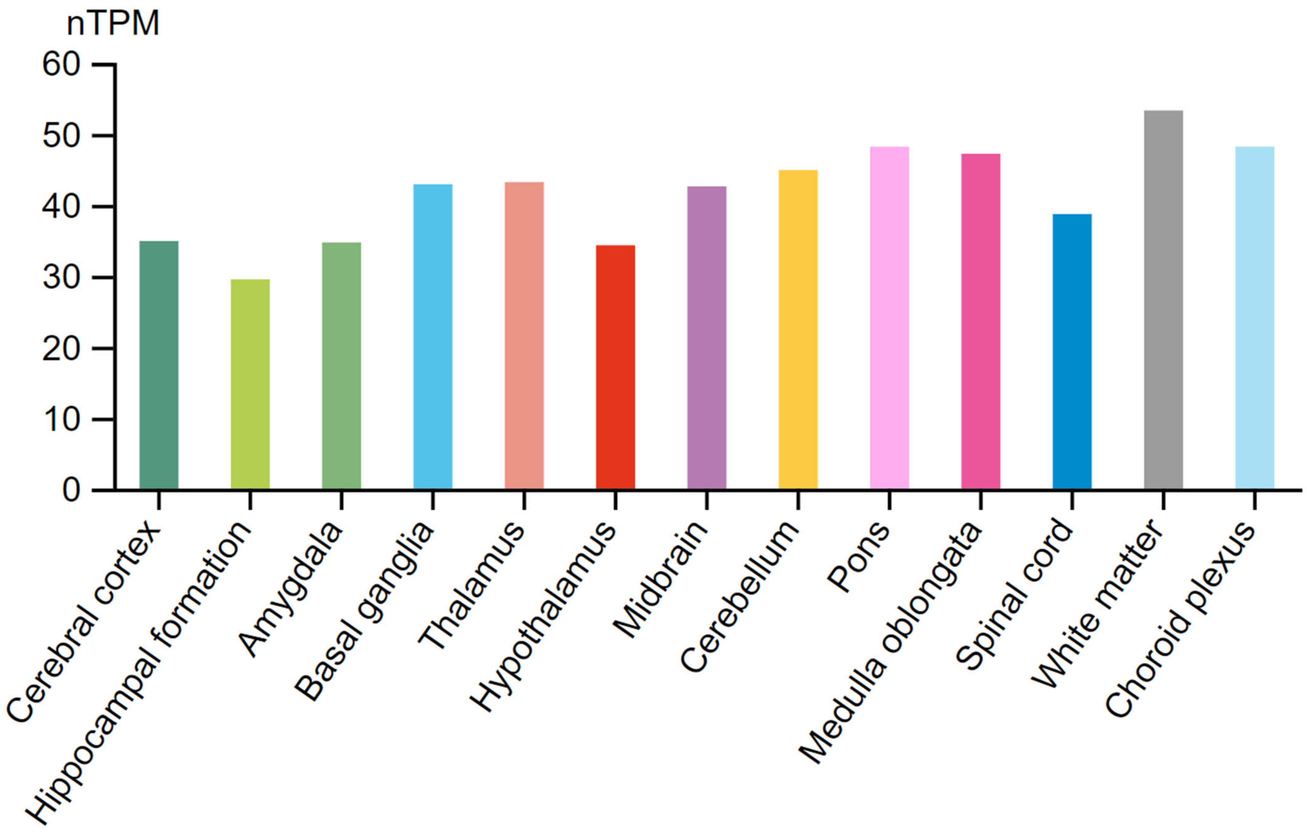

Supplementary Figure 12: RNA expression levels (normalized) for *INSR* in transcripts per million (nTPM) in 13 regions of the human brain from the HPA Human brain dataset (https://www.proteinatlas.org/). *INSR* has the highest expression in the white matter. The height of each bar shows the respective expression in each sub-region.

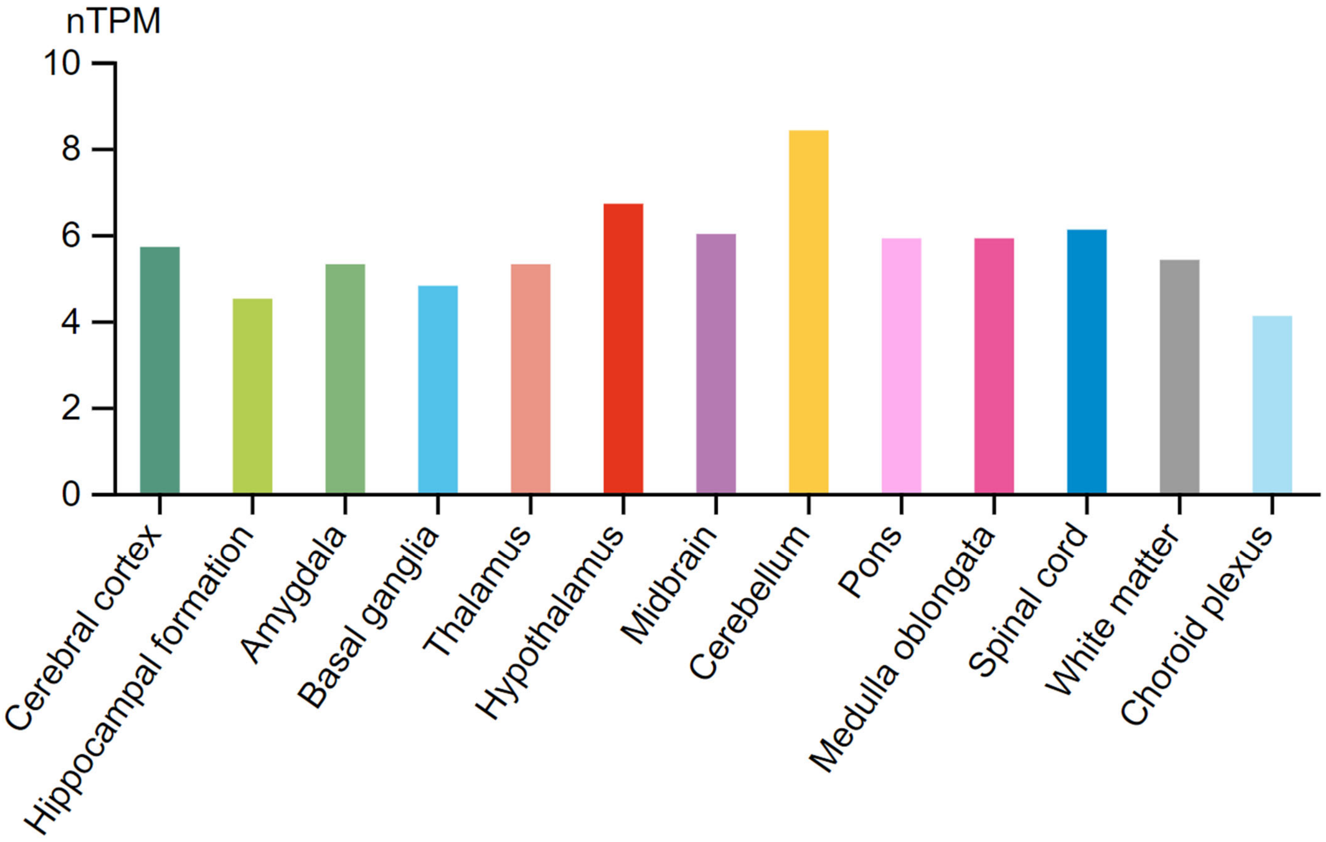

Supplementary Figure 13: The normalized expression levels of *NFXL1* RNA in transcripts per million (nTPM) for 13 regions of the human brain were obtained from the HPA Human brain dataset (https://www.proteinatlas.org/). The highest expression of *NFXL1* was observed in the cerebellum. The height of each bar represents the corresponding expression in each sub-region.

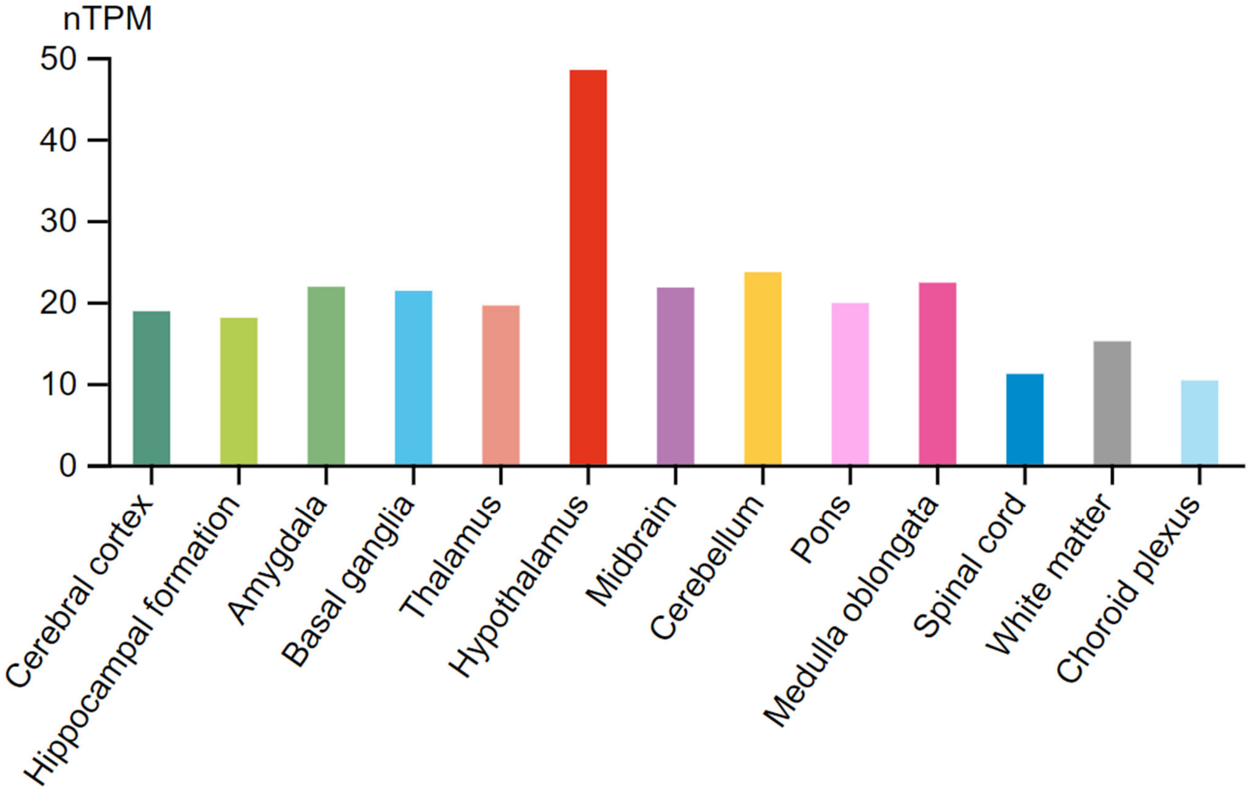

Supplementary Figure 14: The HPA Human brain dataset (https://www.proteinatlas.org/) provided the normalized expression levels of *RYR1* RNA in transcripts per million (nTPM) for 13 regions of the human brain. Hypothalamus exhibited the most prominent expression of *RYR1*. The individual expression in each sub-region is represented by the height of the respective bars.

Supplementary Table 1: Primer sequences for Sanger sequencing, cDNA analyses and allele specific PCR

Primer Name	Sequence	Product Size (bp)	*Comment
INSR_F<br>INSR_R	GTGGAATTCAGTGAGCGACA<br>GTGCCCTTACTCGGACACAT	1001	For Sanger sequencing of *INSR*
INSR_OF<br>INSR_OR	GGGAGGTGCAGAGATGTTTAGTGAGCAA<br>TTCTCTGATTTCTTCCCCTTCCCTTGCT	562	For use in competitive allele PCR as a constant product for *INSR* and providing the Forward or Reverse primers for the specific alleles
INSR_INNF	CACCAGTGCCTGAAGAGGTTTTTCTGTAG**C**	285	Forward primer specific to the mutant *INSR* allele
INSR_INNR	CTGTCCTCAAAGGCGTTGGTTTTGCT**T**	384	Reverse primer specific to the wild-type *INSR* allele
NFXL1_OF<br>NFXL1_OR	CAGATACATGAGCACATACTGTTGGAAAA<br>GAAGAACATGGCACTTTGTTGATGTTTA	500	For Sanger sequencing, also as Tetra primer ARMS PCR constant product for *NFXL1* and providing the Forward or Reverse primers for the specific alleles
NFXL1_INNF	TTATTACAGGAGATGACTAACTTGTCGA**C**	335	Forward primer specific to the *NFXL1* wild-type allele
NFXL1_INNR	TGTCACCGAGGTCCCTGTGAAACCT**A**	220	Reverse primer specific to the *NFXL1* mutant allele
RYRUFO<br>RYRURO	CTCCCTCACCTGAAGCCCCCAGTCCCAG<br>CCACCCCTCAGTACCCTCCTGCACCCCAT	430	Tetra primer ARMS PCR constant product for *RYR1* c.1108G>C variant as well as for providing the Forward or Reverse primers for the specific alleles
RYRUFI	ACCCCAAGGCCCTGCGGCTCGCC**C**	188	Forward primer specific to the mutant *RYR1* c.1108G>C allele
RYRURI	GCTGGGATTACACCCACCTTCTTCTTGAGGA**C**	298	Reverse primer specific to the wild-type *RYR1* c.1108G>C allele
RYRDFO<br>RYRDRO	TGCATAACCCACACCTCCTTCATAATTT<br>AACTTGCAGAACCTTCTCCTCCTTGAAC	385	Tetra primer ARMS PCR constant product for *RYR1* c.7949G>A variant as

			well as for providing the Forward or Reverse primers for the specific alleles
RYRDFI	CAGCTCCTCACCAACCACTATGATC**A**	264	Forward primer specific to the mutant *RYR1* c.7949G>A allele
RYRDRI	GGGTAGGCAGTAGTACTTCCAACCG**C**	173	Reverse primer specific to the wild-type *RYR1* c.7949G>A allele
GAPDH_F^<br>GAPDH_R^	TGCCTCCTGCACCACCAACT<br>TGCCTGCTTCACCACCTT	542	As a housekeeping gene specific product for cDNA
CDFINSRO^<br>CDRINSRO^	ACCTATGGGGCCAAGAGTGACATCATTTAT<br>CCGATAACTCACTTCATACAGCACGATCAG	819 or 855	Forward primer in *INSR* exon 9 and Reverse primer in *INSR* exon 13
CDFINSRI^<br>CDRINSRI^	TCATCCCAGATTATTCTGAAGTGGAAACCA<br>ACGTTGTTCTCAAAGATTTCATGCGTCAC	659 or 695	Forward primer in *INSR* exon 9 and Reverse primer in *INSR* exon 13 (for nested PCR on product from the outer primers)

Underlined nucleotides were deliberately mismatched to increase the specificity of the allele specific reactions, while those shown in bold are allele specific nucleotides. bp-base pair, ^Primers for amplification from cDNA. Two different sized products are expected due to distinct *INSR* isoforms. Nested PCR was completed for *INSR* cDNA analyses to enhance the specificity. This involved amplification with the outer primers followed by re-amplification of the diluted product with the different inner primers. The product obtained after nested PCR was cloned and analyzed as described in the manuscript.

Supplementary Table 2: Compound heterozygous variants in families PSYAK10 and PSYAK22 after exome sequencing data analyses

Family ID	Gene	*gDNA change	RefSeq ID	cDNA change and Amino acid change	dbSNP	Aggregated Allele Frequency (%)	Conservation GERP	Predictions						Comments
						gnomAD		SIFT	PolyPhen2	REVEL	MT	FATHMM	SpliceAI	
PSYAK10	*HEATR 1*	chr1: 236749639 T>C	NM_018 072.6	c.1829A>G p.Asn610Ser Missense	*rs549697437*	0.000147335 (no homozygote)	-9.72	0.37 B	0.01 B	0.04 B	0.00 B	-0.07 U	0.01 B	Predicted mostly benign, amino acid not conserved
	HEATR 1	chr1: 236757378 T>C	NM_018 072.6	c.1127A>G p.Lys376Arg Missense	*rs759296237*	0.0000372 (no homozygote)	3.31	0.52 B	0.01 B	0.09 B	0.00 B	-0.18 U	0.01 B	Predicted mostly benign, amino acid not conserved
	ATP8A 2	chr13: 26273377 G>A	NM_016 529.6	c.2278G>A p.Val760Met Missense	*rs35540339*	0.008706308 (no homozygote)	1.38	0.07 U	0.47 U	0.08 B	0.59 D	-0.05 U	0.00 B	Predicted mostly uncertain or benign, amino

														acid not conserved
	ATP8A2	chr13: 26434393 C>T	NM_016529.6	c.3017C>T p.Thr1006Ile Missense	NA	0	5.85	0.01 U	NA	0.78 D	1 D	-2.37 U	0.00 B	Predicted mostly uncertain or deleterious, amino acid not conserved
PSYAK22	*HMCN2*	chr9: 133301200 C>T	NM_001291815.2	c.12256C>T p.Arg4086Trp Missense	*rs531676404*	0.000933 (no homozygote)	4.68	NA	0.01 B	0.12 B	NA	NA	0.00 B	Predicted benign, amino acid not conserved
	HMCN2	chr9: 133251339 C>T	NM_001291815.2	c.6369C>T p.Leu2123Leu Synonymous	*rs370715218*	0.00009563 (no homozygote)	NA	NA	NA	NA	NA	NA	0.00 B	Not likely to affect splicing
	RPS6KA4	chr11: 64129199 C>T	NM_003942.3	c.737C>T p.Thr246Met Missense	*rs545430280*	0.000164 (no homozygote)	4	0 D	0.905 U	0.17 B	0.959824 D	1.72 U	0.00 B	Predicted uncertain or benign, amino acid not conserved
	RPS6KA4	chr11: 64132817 A>G	NM_003942.3	c.951A>G p.Pro317Pro Synonymous	*rs543314513*	0.000919 (6 homozygotes)	NA	NA	NA	NA	NA	NA	0.00 B	Not likely to affect splicing,

														multiple number of homozygotes in controls
	PRPF39	chr14: 45566100 G>A	NM_017922.4	c.461G>A p.Arg154Gln Missense	*rs41307100*	0.000432 (no homozygote)	5.52	0.314 B	0.77 U	0.06 B	1 D	1.05 U	0.00 B	Predicted mostly uncertain or benign, amino acid not conserved
	PRPF39	chr14: 45571811 A>G	NM_017922.4	c.649A>G p.Asn217Asp Missense	*rs45500394*	0.000559 (1 homozygote)	5.61	0.609 B	0.005 B	0.07 B	0.80078 D	1.42 U	0.00 B	Predicted mostly benign, amino acid not conserved
	LOC400499	Chr16: 11582698 G>C	NM_001370704.1	c.1755C>G p.Ile585Met Missense	*rs987269977*	0	2.07	NA	NA	NA	1 D	NA	0.00 B	Ambiguous predictions, amino acid not conserved
	LOC400499	chr16: 11485908 C>A	NM_001370704.1	c.8491G>T p.Val2831Phe Missense	*rs113210593*	0.000322 (no homozygote)	-8.53	NA	0.001 B	NA	NA	NA	0.00 B	Benign predictions, amino acid not conserved

	CDK12	chr17: 37619100 A>G	NM_016507.4	c.776A>G p.Tyr259Cys Missense	*rs141321265*	0.000147 (no homozygote)	5.17	0 D	NA	0.12 B	0.99 D	0.99 U	0.00 B	Predicted mostly deleterious or uncertain, amino acid conserved
	CDK12	chr17: 37682394 G>A	NM_016507.4	c.3585G>A p.Thr1195Thr Synonymous	*rs140167318*	0.000597 (no homozygote)	NA	NA	NA	NA	NA	NA	0.00 B	Not likely to affect splicing
	LAMA5	chr20: 60911398 G>A	NM_005560.6	c.2321C>T p.Thr774Ile Missense	*rs145721906*	0.001465 (2 homozygotes)	3.74	0.01 U	0.70 U	0.09 B	0.99 D	-0.01 U	0.00 B	Predicted mostly uncertain or benign, amino acid not conserved
	LAMA5	chr20: 60887732 G>A	NM_005560.6	c.9183C>T p.Asp3061Asp Synonymous	*rs145862467*	0.000816 (no homozygote)	NA	0.01 U	NA	NA	NA	NA	0.00 B	Not likely to affect splicing
	PLXNB2	Chr22: 50719237 G>A	NM_012401.4	c.3929C>T p.Pro1310Leu Missense	*rs201776829*	0.00101 (2 homozygotes)	0.739	0.024 U	0.007 B	0.037 B	0.002219	2.79 U	0.00 B	Predicted mostly benign or uncertain, amino acid not conserved

	PLXNB 2	Chr22: 50717291 C>T	NM_012 401.4	c.4539G>A p.Val1513Val Synonymous	*rs186846228*	0.000317 (no homozygote)	2.33	NA	NA	NA	1 D	NA	0.00 B	Not likely to affect splicing

*Variant position according to Human Feb.2009 (GRCh37/hg19) Assembly. RefSeq= Reference Sequence, gnomAD= Genome Aggregation Database, GERP= Genomic Evolutionary Rate Prediction (negative and low scores indicate no or low conservation), SIFT=Sorting Intolerant from Tolerant algorithm, REVEL=Rare Exome Variant Ensemble Learner, MT=Mutation Taster, FATHMM=Functional Annotation Through Hidden Markov Models, SpliceAI=Splice Altering algorithm, D=Damaging or Deleterious, T=Tolerated, NA=Not applicable, B=Benign, U=Uncertain.

Supplementary Table 3: Rare segregating homozygous/hemizygous variants for families PSYAK1, PSYAK4, PSYAK5, PSYAK6, PSYAK7 and PSYAK8 after exome sequencing data analyses

Family ID	Gene	*gDNA change	RefSeq ID	cDNA change and Amino acid change	dbSNP	Aggregated Allele Frequency (%)	Conservation	Predictions						Comments
						gnomAD	GERP	SIFT	PolyPhen2	REVEL	MT	FATHMM	SpliceAI	
PSYAK1	*APOO*	chrX: 23897140 G>A	NM_024122.4	c.129C>T p.Tyr43Tyr Synonymous	*rs566251924*	0.000246 (34 hemizygotes)	NA	NA	NA	NA	NA	NA	0.1 U	Not likely to affect splicing, multiple number of hemizygotes in controls
	TREX2	chrX: 152710442 C>T	NM_080701.3	c.447G>A p.Pro149Pro Synonymous	*rs782443247*	0.000433 (23 hemizygotes)	NA	NA	NA	NA	NA	NA	0.00 B	Not likely to affect splicing, multiple number of hemizygotes in controls

PSYAK4	*CGNL1*	chr15: 57754087 C>T	NM_032866.4	c.2400C>T p.Thr800Thr Synonymous	*rs769424031*	0.0000279 (no homozygote)	NA	NA	NA	NA	NA	NA	0.00 B	Not likely to affect splicing
PSYAK5	*IL12RB2*	chr1: 67787302 G>A	NM_001559.2	c.94G>A p.Asp32Asn Missense	*rs775778071*	0.0000399 (no homozygote)	3.24	0.312T	0.631 P	0.18 B	0 N	-1.17 T	0.01 B	Predicted mostly tolerated/benign, amino acid not conserved
	CCDC140	chr2: 223168774 G>A	NM_153038.1	c.153G>A p.Gln51Gln Synonymous	*rs140626793*	0.0004854 (2 homozygotes)	NA	NA	NA	NA	NA	NA	0.00 B	Not likely to affect splicing
	ILKAP	chr2: 239092306 G>A	NM_030768.3	c.702C>T p.Leu234Leu Synonymous	*rs759797592*	0.00000824 (no homozygote)	NA	NA	NA	NA	NA	NA	0.00 B	Not likely to affect splicing
	PDGFRB	chr5: 149515433 G>A	NM_002609.4	c.49C>T p.Leu17Leu Synonymous	NA	0	NA	NA	NA	NA	NA	NA	0.06 B	Not likely to affect splicing
	KIF4B	chr5: 154393620 A>G	NM_001099293.2	c.201A>G p.Lys67Lys Synonymous	*rs766518850*	0.0000318 (no homozygote)	NA	NA	NA	NA	NA	NA	0.00 B	Not likely to affect splicing

	RBM20	chr10: 112572243 C>T	NM_001134363.3	c.2088C>T p.Asn696Asn Synonymous	*rs1349172728*	0.0000061 (no homozygote)	NA	NA	NA	NA	NA	NA	0.00 B	Not likely to affect splicing
	BTBD16	chr10: 124091988 T>G	NM_144587.3	c.1124T>G p.Leu375Arg Missense	*rs750215493*	0.0000877 (no homozygote)	4.68	0.006D	0.986 D	0.24 B	0 N	1.65 T	0.15 B	Predicted uncertain, amino acid not conserved
	DOCK1	chr10: 128797664 A>C	NM_001290223.1	c.814A>C p.Ile272Leu Missense	*rs777117151*	0.0003463 (no homozygote)	3.97	0.275T	0.031 B	0.27 B	0.999 D	2.25 T	0.01 B	Predicted mostly tolerated/benign, amino acid conserved
	RBM23	chr14: 23377546 G>A	NM_001077351.2	c.223C>T p.Arg75Cys Missense	*rs1168156871*	0	3.93	0D	NA	0.31 U	1 D	-1.33 T	0.06 B	Predicted mostly tolerated/benign, amino acid not conserved
	LTB4R2	chr14: 24780751 A>G	NM_019839.5	c.881A>G p.Asp294Gly Missense	*rs199950899*	0.0008573 (1 homozygote)	4.63	0.763T	0.156 B	0.17 B	1 D	-0.36 T	0.01 B	Predicted mostly tolerated/benign, amino

														acid not conserved
	ADRM1	chr20: 60883129 C>T	NM_007002.4	c.909C>T p.Asn303Asn Synonymous	*rs756225724*	0.0001296 (no homozygote)	NA	NA	NA	NA	NA	NA	0.01 B	Not likely to affect splicing
	LAMA5	chr20: 60887058 G>T	NM_005560.5	c.9553C>A p.Leu3185Ile Missense	NA	0	-3.47	0.93 T	NA	0.12 B	0 N	-1.16 T	0.14 B	Predicted tolerated/benign, amino acid not conserved
	LAMA5	chr20: 60911480 G>A	NM_005560.5	c.2239C>T p.Arg747Trp Missense	*rs370940497*	0.00012445 (no homozygote)	4.91	0.005 D	1 D	0.62 U	1 D	-0.12 T	0.00 B	Predicted uncertain, amino acid not conserved
	ARVCF	chr22: 19964990 G>A	NM_001670.3	c.1818C>T p.Ser606Ser Synonymous	NA	0	NA	NA	NA	NA	NA	NA	0.00 B	Not likely to affect splicing
	SCARF2	chr22: 20780337 G>A	NM_182895.4	c.1926C>T p.Gly642Gly Synonymous	*rs768694133*	0.0000190 (no homozygote)	NA	NA	NA	NA	NA	NA	0.00 B	Not likely to affect splicing
	GGT5	chr22: 24628842 C>T	NM_004121.3	c.545G>A p.Arg182His Missense	*rs578189665*	0.0002071 (no homozygote)	-3.56	0.227T	0.005 B	0.01 B	0 N	3.21 T	0.00 B	Predicted benign, amino acid not conserved

PSYAK5	*CRYBB3*	chr22: 25601329 C>T	NM_004076.5	c.470C>T p.Thr157Met Missense	*rs375467933*	0.0001799 (no homozygote)	4.14	0.007D	0.998 D	0.69 D	1 D	-1 T	0.01 B	Four deleterious predictions but methionine is the wild type amino acid in many mammals
	ASPHD2	chr22: 26839059 C>A	NM_020437.5	c.1001-4C>A splicing	*rs75079091*	0.0018455 (6 homozygotes)	NA	NA	NA	NA	NA	NA	0.00 B	Not likely to affect splicing
PSYAK6	*MSR1*	chr8: 15977955 G>A	NM_138715.3	c.1194C>T p.Ala398Ala Synonymous	*rs562625597*	0.001106 (7 homozygotes)	NA	NA	NA	NA	NA	NA	0.00 B	Not likely to affect splicing
	MLLT6	chr17: 36878264 C>T	NM_005937.3	c.2576C>T p.Pro859Leu Missense	*rs756140181*	0.0000355 (no homozygote)	2.47	NA	NA	o.o5 B	0.951 D	NA	0.00 B	Predicted benign, amino acid not conserved
	KRT23	chr17: 39081730 G>A	NM_015515.4	c.1018C>T p.Leu340Leu Synonymous	*rs148318927*	0.003118	NA	NA	NA	NA	NA	NA	0.00 B	Not likely to affect splicing

						(4 homozygotes)								
	KRT37	chr17: 39579096 G>A	NM_003 770.4	c.666C>T p.Ala222Ala Synonymous	*rs559004 919*	0.0000836 (no homozygote)	NA	NA	NA	NA	NA	NA	0.00 B	Not likely to affect splicing
	CFAP9 7D1	chr17: 41861265 T>G	NM_001 136483. 2	c.495+4T>G Splicing	*rs148379 784*	0.000763 (no homozygote)	NA	NA	NA	NA	NA	NA	0.04 B	Not likely to affect splicing
	BCAR3	chr1: 94054736 G>T	NM_003 567.4	c.727C>A p.Gln243Lys Missense	*rs778087 104*	0.000028 (1 homozygote)	4.96	0.13 9 B	0.00 2B	0.03 B	0.86 0 D	0.06 U	0.00 B	Predicted benign, amino acid not conserved
	MTR	chr1: 236992561 A>T	NM_000 254.2	c.1068A>T p.Leu356Phe Missense	NA	0	1.46	0.05 5 U	NA	0.14 B	0.97 7 D	1.2 U	0.00 B	Predicted benign, amino acid not conserved
	RYR2	chr1: 237730032 A>G	NM_001 035.3	c.3380A>G p.Glu1127Gly Missense	*rs200525 962*	0.0010793 (5 homozygotes)	5.29	0.01 4U	0.99 9D	0.50 U	0.99 9 D	-0.43 U	0.00 B	Predicted benign, amino acid not conserved
PSYAK7	*USP38*	chr4: 144109093 G>A	NM_032 557.6	c.797G>A p.Ser266Asn Missense	*rs771553 760*	0.0000558 (no homozygote)	4.35	0.66 B	0.02 B	0.06 B	0.99 9 D	-0.29 U	0.00 B	Predicted mostly benign,

														amino acid not conserved
	OAS1	chr12: 113346509 G>A	NM_016816.4	c.349G>A p.Val117Met Missense	*rs200127558*	0.000131 (no homozygote)	2.46	0.012 U	1D	0.13 B	0N	1.76 U	0.0 B	Predicted mostly uncertain or benign, amino acid conserved
PSYAK8	*PPP1R27*	chr17: 79792384 T>C	NM_001007533.4	c.336A>G p.Ile112Met Missense	*rs201778865*	0.000994 (no homozygote)	-5.21	0D	0.985 D	0.38 U	0.885983 D	-0.3 U	0.0 B	Predicted mostly uncertain or benign, amino acid not conserved

*Variant position according to Human Feb.2009 (GRCh37/hg19) Assembly. RefSeq= Reference Sequence, gnomAD= Genome Aggregation Database, GERP= Genomic Evolutionary Rate Prediction (negative and low scores indicate no or low conservation), SIFT=Sorting Intolerant from Tolerant algorithm, REVEL=Rare Exome Variant Ensemble Learner, MT=Mutation Taster, FATHMM=Functional Annotation Through Hidden Markov Models, SpliceAI=Splice Altering algorithm, D=Damaging or Deleterious, T=Tolerated, NA=Not applicable, B=Benign, U=Uncertain.

Supplementary Table 4: Compound heterozygous variants in families PSYAK1, PSYAK4, PSYAK6 and PSYAK8 after exome sequencing data analyses

Family ID	Gene	*gDNA change	RefSeq ID	cDNA change and Amino acid change	dbSNP	Aggregated Allele Frequency (%)	Conservation GERP	Predictions						Comments
						gnomAD		SIFT	PolyPhen2	REVEL	MT	FATHMM	SpliceAI	
PSYAK1	*MASP1*	chr3: 186943280 G>A	NM_001 879.5	c.1573C>T p.Arg525Trp Missense	*rs28945073*	0.000612 (4 homozygotes)	2.95	0.00 5 U	0.84 U	0.32 U	0.0 B	-2.5 U	0.00 B	Predicted mostly uncertain or benign, amino acid not conserved
	MASP1	chr3: 186978566 G>A	NM_139 125.3	c.510C>T p.Phe170Phe Synonymous	*rs553549384*	0.000629 (no homozygote)	NA	NA	NA	NA	NA	NA	0.01 B	Not likely to affect splicing, common in internal unrelated individuals' exome data

	TBC1D 16	chr17: 77984150 G>A	NM_019 020.4	c.588C>T p.Thr196Thr Synonymous	*rs536902994*	0.000176 (1 homozygote)	NA	NA	NA	NA	NA	NA	0.01 B	Not likely to affect splicing
	TBC1D 16	chr17: 77984468 C>T	NM_019 020.4	c.270G>A p.Gln90Gln Synonymous	*rs376202586*	0.00126 (2 homozygotes)	NA	NA	NA	NA	NA	NA	0.01 B	Not likely to affect splicing
	DLL4	chr15: 41222855 C>T	NM_019 074.4	c.369C>T p.His123His Synonymous	*rs187700175*	0.004114 (14 homozygotes)	NA	NA	NA	NA	NA	NA	0.01 B	Not likely to affect splicing, multiple number of homozygotes in controls
	DLL4	chr15: 41228616 G>A	NM_019 074.4	c.1431G>A p.Val477Val Synonymous	*rs540754224*	0.000245 (no homozygote)	NA	NA	NA	NA	NA	NA	0.01 B	Not likely to affect splicing
	ENTPD 6	chr20: 25205945 G>A	NM_001 247.4	c.1348G>A p.Val450Met Missense	*rs775848233*	0.0000120 (no homozygote)	4.48	0.10 B	0.39 U	0.10 B	0.63 D	2.78 U	0.00 B	Predicted benign, amino acid not conserved
	ENTPD 6	chr20: 25176504 G>A	NM_001 247.4	c.-16+1G>A Splicing	*rs758504154*	0.000236 (no homozygote)	-0.81	NA	NA	NA	0.01 B	NA	0.99 D	Predicted to affect splicing of an alternative

														exon. This nucleotide is not conserved in evolution
	A4GNT	chr3: 137843541 C>T	NM_016161.2	c.588G>A<br>p.Gly196Gly<br>Synonymous	*rs563180536*	0.0000477 (no homozygote)	NA	NA	NA	NA	NA	NA	0.00<br>B	Not likely to affect splicing
	A4GNT	chr3: 137843632 A>G	NM_016161.2	c.497T>C<br>p.Met166Thr<br>Missense	*rs1398875186*	0.00000397 (no homozygote)	5.27	0.002<br>U	NA	0.85<br>D	1<br>D	-0.33<br>U	0.00<br>B	Predicted deleterious by two software, amino acid not conserved
	TTN	chr2: 179432210 C>T	NM_001267550.2	c.78649G>A<br>p.Val26217Ile<br>Missense	NA	0	5.65	0.14<br>B	NA	0.22<br>B	1<br>D	-0.62<br>U	0.00<br>B	Predicted mostly benign, amino acid not conserved
	TTN	chr2: 179610985 C>T	NM_133379.4	c.16142G>A<br>p.Arg5381His<br>Missense	*rs369189673*	0.00035 (no homozygote)	5.88	0.37<br>B	1<br>D	0.21<br>B	1<br>D	-0.29<br>U	0.00<br>B	Predicted mostly benign, amino acid not conserved

	CENPX	chr17: 79977489 C>G	NM_001330536.1	c.170G>C p.Gly57Ala Missense	*rs540001749*	0.0000940 (no homozygote)	-1.55	NA	0.01 B	NA	0.01 B	NA	0.02 B	Predicted mostly benign, amino acid not conserved
	CENPX	chr17: 79977559 C>T	NM_001271006.2	c.100G>A p.Ala34Thr Missense	*rs540391402*	0.000991 (1 homozygote)	1.60	0.01 U	0.87 U	0.29 U	1 D	NA	0.00 B	Predicted mostly uncertain or benign, amino acid not conserved
PSYAK4	*KIDINS 220*	chr2: 8958931 A>G	NM_020738.3	c.109-8T>C Splicing	*rs536316090*	0.000293 (no homozygote)	NA	NA	NA	NA	NA	NA	0.00 B	Predicted benign, not likely to affect splicing
	KIDINS 220	chr2: 8877136 A>G	NM_020738.3	c.3586-7T>C Splicing	*rs139887022*	0.003086 (6 homozygotes)	NA	NA	NA	NA	NA	NA	0.00 B	Predicted benign, not likely to affect splicing, multiple number of homozygotes in controls

PSYAK6	*TTN*	chr2: 179422413 T>C	NM_001 267550. 2	c.87668A>G p.His29223Ar g Missense	*rs369880812*	0.0000040 (no homozygote)	5.66	0.07 U	0.23 U	0.35 U	1 D	0.66 U	0.00 B	Predicted mostly uncertain or benign, amino acid conserved
	TTN	chr2: 179532362 T>C	NM_001 267550. 2	c.35629+3A> G Splicing	*rs751928755*	0.0000087 (no homozygote)	NA	NA	NA	NA	NA	NA	0.79 D	Predicted to affect splicing, nucleotide not conserved in one species
	ADGB	chr6: 147045402 A>G	NM_024 694.4	c.2176A>G p.Ser726Gly Missense	NA	NA	0.39	0.23 B	NA	0.02 B	0.00 B	0.43 U	0.01 B	Predicted mostly benign, amino acid not conserved
	ADGB	chr6: 146985454 A>G	NM_024 694.4	c.730A>G p.Ile244Val Missense	*rs1419108806*	0.0000130 (no homozygote)	4.23	0.02 B	NA	0.18 B	0.53 D	0.99 U	0.00 B	Predicted mostly benign, amino acid not conserved

	DOP1B	chr21: 37618180 A>G	NM_001320714.1	c.3902A>G p.Gln1301Arg Missense	*rs201825608*	0.0006448 (no homozygote)	4.62	0.02 U	0.35 U	0.13 B	0.99 D	0.90 U	0.00 B	Predicted mostly benign, amino acid not conserved
	DOP1B	chr21: 37665836 C>T	NM_001320714.1	c.6864C>T p.Ile2288Ile Synonymous	*rs61752552*	0.001030 (2 homozygotes)	NA	NA	NA	NA	NA	NA	0.00 B	Not likely to affect splicing
PSYAK8	*CFAP100*	Chr3: 126132967 C>G	NM_182628.3	c.170C>G p.Ser57Cys Missense	NA	NA	4.60	0.0 D	NA	0.09 B	0.00 B	1.44 U	0.06 B	Predicted mostly benign, amino acid not conserved
	CFAP100	chr3: 126139046 C>A	NM_182628.3	c.1056C>A p.Ser352Arg Missense	*rs368926593*	0.0000281 (no homozygote)	-6.12	0.17 B	0.09 B	0.08 B	0.01 B	1.38 U	0.00 B	Predicted mostly benign, amino acid not conserved
	RYR1	**Chr19: 38939439 G>C**	**NM_000540.2**	**c.1108G>C p.Val370Leu Missense**	***rs537344365***	**0.000095 (no homozygote)**	**3.50**	**0.14 B**	**0.17 U**	**0.27 B**	**0.01 B**	**-2.75 U**	**0.00 B**	**Predicted uncertain or benign, amino acid**

														**not conserved**
	RYR1	**chr19: 38994882 G>A**	**NM_000 540.2**	**c.7949G>A p.Arg2650Hi s Missense**	***rs145733990***	**0.00000399 (no homozygote)**	**4.04**	**0.01 U**	**0.98 U**	**0.80 D**	**1 D**	**-4.51 D**	**0.00 B**	**Predicted mostly deleterious or uncertain, amino acid conserved**

*Variant position according to Human Feb.2009 (GRCh37/hg19) Assembly. RefSeq= Reference Sequence, gnomAD= Genome Aggregation Database, GERP= Genomic Evolutionary Rate Prediction (negative and low scores indicate no or low conservation), SIFT=Sorting Intolerant from Tolerant algorithm, REVEL=Rare Exome Variant Ensemble Learner, MT=Mutation Taster, FATHMM=Functional Annotation Through Hidden Markov Models, SpliceAI=Splice Altering algorithm, D=Damaging or Deleterious, T=Tolerated, NA=Not applicable, B=Benign, U=Uncertain.

Supplementary references

1. Shaffer, D.; Fisher, P.; Lucas, C. P.; Dulcan, M. K.; Schwab-Stone, M. E., NIMH Diagnostic Interview Schedule for Children Version IV (NIMH DISC-IV): description, differences from previous versions, and reliability of some common diagnoses. *J Am Acad Child Psychiatry* **2000,** 39, (1), 28-38.
2. Folstein, M. F.; Folstein, S. E.; McHugh, P. R., "Mini-mental state": a practical method for grading the cognitive state of patients for the clinician. *J Psychiatr Res* **1975,** 12, (3), 189-198.
3. Hamilton, M., Rating depressive patients. *J Clin Psychiatry* **1980,** 41, (12), 21-24.
4. Semple, R., Diagnostic Interview for Psychosis and Affective Disorders (DI-PAD). *Los Angeles: University of Southern California* **2008**
5. Kay, S. R.; Opler, L. A.; Spitzer, R. L.; Williams, J. B.; Fiszbein, A.; Gorelick, A., SCID-PANSS: two-tier diagnostic system for psychotic disorders. *Compr. Psychiatry* **1991,** 32, (4), 355-361.
6. Carr, I. M.; Bhaskar, S.; O'Sullivan, J.; Aldahmesh, M. A.; Shamseldin, H. E.; Markham, A. F.; Bonthron, D. T.; Black, G.; Alkuraya, F. S., Autozygosity mapping with exome sequence data. *Hum Mutat* **2013,** 34, (1), 50-56.

7. Kanwal, A.; Sheikh, S. A.; Iftikhar, A.; Naz, S.; Pardo, J. V., Preliminary studies on apparent mendelian psychotic disorders in consanguineous families. *BMC Psychiatr* **2022b,** 22, (1), 709.
